# Probabilistic associative learning suffices for learning the temporal structure of multiple sequences

**DOI:** 10.1371/journal.pone.0220161

**Published:** 2019-08-01

**Authors:** Ramon H. Martinez, Anders Lansner, Pawel Herman

**Affiliations:** 1 Computational Brain Science Lab, KTH Royal Institute of Technology, Stockholm, Sweden; 2 Mathematics Department, Stockholm University, Stockholm, Sweden; Research Center Jülich, GERMANY

## Abstract

From memorizing a musical tune to navigating a well known route, many of our underlying behaviors have a strong temporal component. While the mechanisms behind the sequential nature of the underlying brain activity are likely multifarious and multi-scale, in this work we attempt to characterize to what degree some of this properties can be explained as a consequence of simple associative learning. To this end, we employ a parsimonious firing-rate attractor network equipped with the Hebbian-like Bayesian Confidence Propagating Neural Network (BCPNN) learning rule relying on synaptic traces with asymmetric temporal characteristics. The proposed network model is able to encode and reproduce temporal aspects of the input, and offers internal control of the recall dynamics by gain modulation. We provide an analytical characterisation of the relationship between the structure of the weight matrix, the dynamical network parameters and the temporal aspects of sequence recall. We also present a computational study of the performance of the system under the effects of noise for an extensive region of the parameter space. Finally, we show how the inclusion of modularity in our network structure facilitates the learning and recall of multiple overlapping sequences even in a noisy regime.

## 1 Introduction

From throwing spears in the savanna to the performance of a well rehearsed dance, human behavior reflects an intrinsic sequential structure. In this light, is not surprising that sequential activity has been found in the neural dynamics across different anatomical brain areas such as the cortex [1–4], the basal ganglia [2, 5–10], the hippocampus [11–15] and the HVC area in songbirds [16, 17]. Moreover, sequential activity is not only present in a wide range of neuroanatomical areas but is also associated with an ample repertoire of behaviors and cognitive processes including sensory perception [18, 19], memory [20–22], motor behavior [23, 24] and decision making [3, 25]. In our view, the entanglement of sequential activity with cognitive processes and behavior strongly suggests that sequential activity is an essential component of the information processing capabilities of the brain and therefore demands better understanding. A plausible hypothesis for the ubiquity of sequential activity is a common learning mechanism for the construction of temporal representations at the network level. Inspired by experimental evidence we propose the following constraints and properties for the neural representations and the underlying network mechanisms: First, the recall dynamics of a sequence should reflect key temporal features of the input or training signal [26]. Second, the network should enable temporal scaling, that is, once a sequential representation has been learned, internal neural network’s mechanisms should suffice to contract or dilate its recall duration [27, 28]. Finally, as the same neural network circuits have been observed to exhibit many sequential trajectories accounting for different behaviors [12], it is desirable for the network to posses mechanisms to store and recall multiple and, to some extent, overlapping sequences [29].

There is evidence that sequential activity can be characterized as a succession of meta-stable cell assemblies in the cortex [21]. Attractor neural networks have a long standing tradition as models of sequential activity with meta-stable states corresponding to attractor patterns [30, 31]. Hopfield in his seminal work [32, 33] already noted that an asymmetric connectivity in a recurrent attractor network was conducive to sequential recall. However, in the most basic implementation, the asynchronous update dynamics of these Hopfield models resulted in mixed patterns, thereby gradually diluting sequential recall with time [34]. To overcome such limitations, temporal traces of the activity were utilized successfully as a mechanism to keep the meta-stable states active for long enough to ensure a successful transition between the patterns and some models even allow for temporal rescaling of the dynamics [35, 36]. However, such models are unable to properly integrate the temporal structure of the input due to the discrete nature of their learning rule. A more sophisticated approach relies on systematically considering all the possible delays of the input and calculate all the resulting cross-correlations [37, 38]. While in principle these models are able to learn arbitrary variations in the temporal structure of the input, in practice they are limited by an explosion in the number of parameters as the connectivity matrix scales with the size of the longest transition. In this work we propose an attractor model that uses the following properties to overcome the aforementioned problems: 1) It exploits temporal traces for learning in a probabilistic framework [39]. The temporal nature of the traces allows us to capture the temporal structure of the input, while avoiding an explosion in the number of parameters by collapsing the temporal structure into statistical estimates of the connectivity. 2) The sequence transition mechanism rests on the meta-stability of the attractor dynamics by means of intrinsic adaptation of the network units coupled with a competition mechanism that biases the transition in the correct direction. At the same time the intrinsic adaptation allows for the internal control and rescaling of the recall dynamics. 3) The use of a modular structure in our network facilitates both flexible learning and recall of overlapping representations.

Several network models have been proposed to account for sequential activity. While Veliz-Cuba et al [40] reported that their network could learn the temporal structure of the input, it required a fine-tuned relationship between synaptic, dynamic and homeostatic parameters. Additionally, their model lacked a mechanism for temporal rescaling and the question of learning multiple sequences was not addressed. In a more recent approach by Pereira and Brunel (2018) [41], persistent or sequential activity dynamics could be learned depending on the temporal structure of the input. However, the proposed network did not solve the problem of temporal scaling nor the acquisition of multiple sequences. Using spike-time-dependent plasticity (STDP) with heterosynaptic competition Fiete et al. [42] demonstrated the capability of their model to learn multiple sequences from random activity but handling input with specific temporal structure was not elaborated in their work. Furthermore, Byrnes et Al [43] addressed the problem of learning overlapping sequences but their approach did not scale well as it relied on a single unique representation for every sequence even if they had overlapping elements. Finally, Murray et al. [44] proposed an inhibitory network inspired by the basal ganglia that achieves temporal rescaling by means of the interplay between synaptic fatigue and external input. In this model, however, the problem of handling multiple sequences could be solved only by assuming the existence of such representations in an upstream network, which we consider as a strongly limiting factor.

Inspired by our previous modelling efforts to study sequence [39] and word list learning phenomena [45] we propose here a modular attractor memory neural network model that learns sequential representations by means of the combination of the Bayesian Confidence Propagating Neural Network (BCPNN) learning mechanism [46] and asymmetrical temporal synaptic traces. We proceed by first presenting the network and its dynamics. Then, we derive analytical formulae for the temporal structure of the recall process in noiseless conditions. We also describe how learning is accomplished in the network through the use of synaptic traces and study how the temporal structure of the input is accounted for in the recall dynamics by means of the BCPNN learning rule. We follow up with a systematic characterization of the effects of noise on the sequence recall capability of the network. Finally, we elaborate on how the modularity of the network enables learning overlapping sequences and discuss key limitations.

## 2 Results

### 2.1 Sequence recall

**Fig 1:**
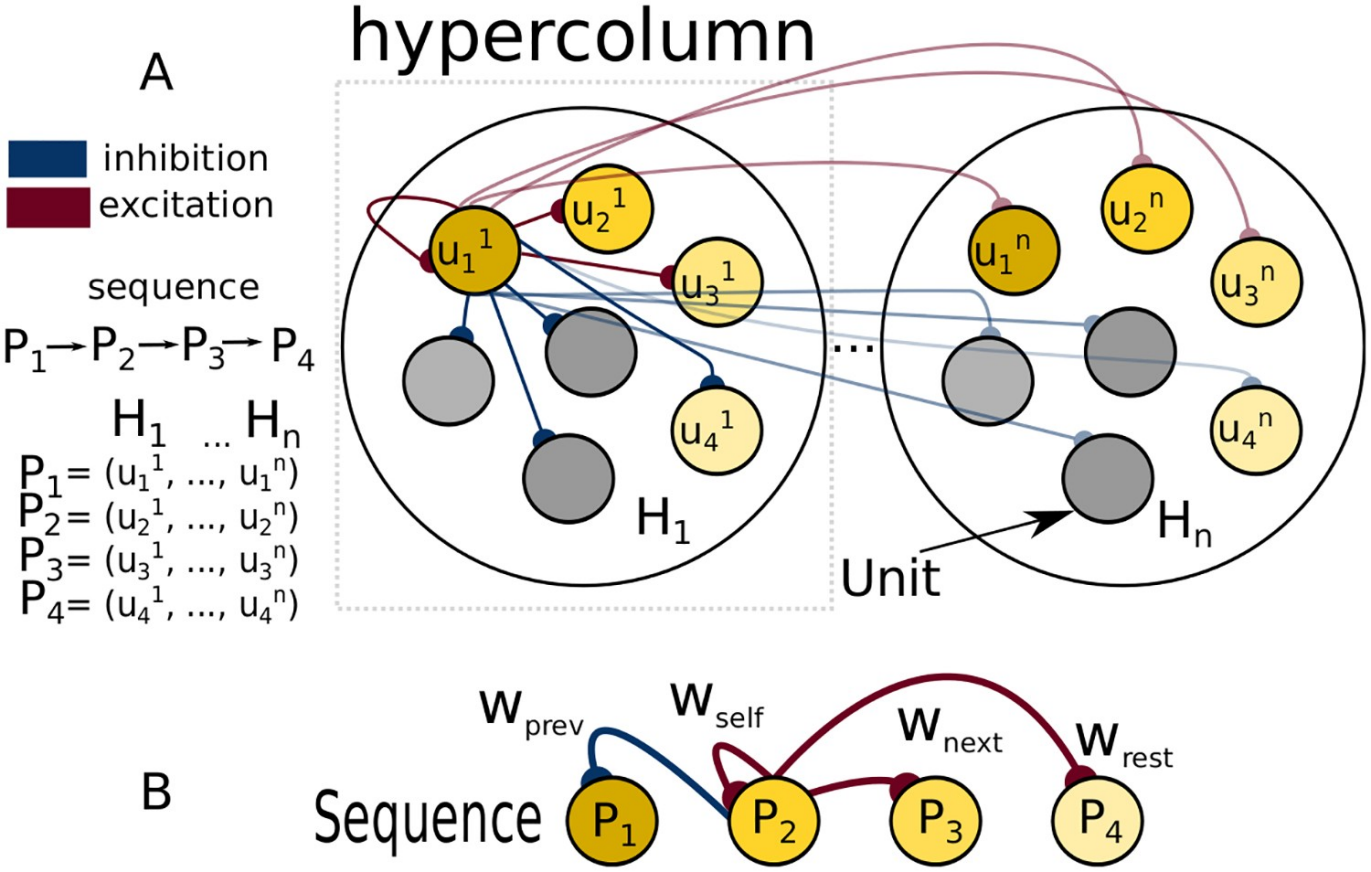
Network architecture and connectivity underlying sequential pattern activation. Network architecture and connectivity underlying sequential pattern activation. (A) network topology. Units $u_{i}^{j}$ are organized into hypercolumns *h*_1_, …, *h*_*H*_. At each point in time only one unit per hypercolumn is active due to a WTA mechanism. Each memory pattern is formed by a set of *H* recurrently connected units distributed across hypercolumns. For simplicity and without compromising the generality we adopt the following notation for patterns $P_{1}=(u_{1}^{1},\ldots ,u_{1}^{H})$. We depict stereotypical network connectivity by showing all the units that emanate from unit $u_{1}^{1}$. The unit has excitatory projections to the proximate units in the sequence (connections from $u_{1}^{1}$ to $u_{2}^{1}$ and $u_{3}^{1}$ and the corresponding units in other hypercolumns) and inhibitory projections to both the units that are farther ahead in the sequence ($u_{1}^{1}$ to $u_{4}^{1}$) and the units that are not in the sequence at all (gray units). (B) abstract representation of the relevant connectivity for sequence dynamics. Please note that only connections from *P*_2_ are shown.

Following previous work on cortical attractor memory modelling [39, 45] we present here a network capable of learning, recalling and processing sequential activity. We utilize a population model of the cortex where each unit represents a population of excitatory neurons in the superficial layer of a cortical column. Consistently with the mesoscale neuroanatomical organization, those units are organized into hypercolumns, where a winner-takes-all (WTA) mechanism representing lateral inhibition keeps the activity within the hypercolumnar module normalized [47]. The topological organization of the model is presented in Fig 1A. The circuit implements attractor dynamics [48] that leads the evolution of the network towards temporary or permanent patterns of activity (pattern refers to a particular collection of active units in the network, see Fig 1A). We refer to these stable or meta-stable states as the stored patterns of the network. The patterns themselves are defined by self-recurrent excitatory connectivity that tends to maintain the pattern in place once activated (represented by *w*_*self*_ in Fig 1B). The patterns can naturally be thought of as cell assemblies distributed among the hypercolumns in the network. The WTA mechanism renders the activity of the units mutually exclusive within the hypercolumns and therefore ensures sparse activity [49]. Sequential activation of patterns can be induced by feed-forward excitation (represented by *w*_*next*_ in Fig 1B) coupled with an adaptation mechanism whose role is to cease current pattern activity thereby counteracting the pattern retention effects of the self-recurrent connectivity.

We model the dynamics of the units with a population model equation [50]. As described in Eq 1 the current *s* changes according to the base rate *β*_*j*_ (also called the bias term) plus the total incoming current from all other N units, $\frac{1}{H}\sum_{i}^{N}w_{ij}o_{i}$, normalized by the number of hypercolumns H. The binary activation variable *o*_*j*_ represents unit activation and is related to the current through a WTA mechanism implemented with a max operation as in Eq 2. This mechanism selects the unit receiving the maximum current at each hypercolumn and activates it. We introduce intrinsic adaptation as a mechanism controlled by the variable *a* in Eq 3 to induce pattern deactivation. *dξ* represents additive white noise with variance *σ*. An extra current *I*_*j*_(*t*) is used to model external input into the system. For the sake of generality, it is important to stress that our current based population model is equivalent to a rate-based formalism, as shown in [51].

$$\begin{array}{cc} \tau_{s}\frac{ds_{j}}{dt} & =\beta_{j}+\frac{1}{H}\sum_{i}^{N}w_{ij}o_{i}-g_{a}a_{j}-s_{j}+\sigma d\xi_{j}+I_{j} \end{array}$$ (1)

$$\begin{array}{c} o_{j}=\{\begin{array}{cc} 1, & s_{j}=\underset{hypercolumn}{\text{max}}\left(s\right),\\ 0, & \text{otherwise} \end{array} \end{array}$$ (2)

$$\begin{array}{cc} \tau_{a}\frac{da_{j}}{dt} & =o_{j}-a_{j} \end{array}$$ (3)

It has long been recognized that an attractor model with asymmetric connectivity produces sequential dynamics [52]. In that vein, we explain now how an asymmetric connectivity matrix coupled with the dynamics of our model brings about sequential activity.

**Fig 2:**
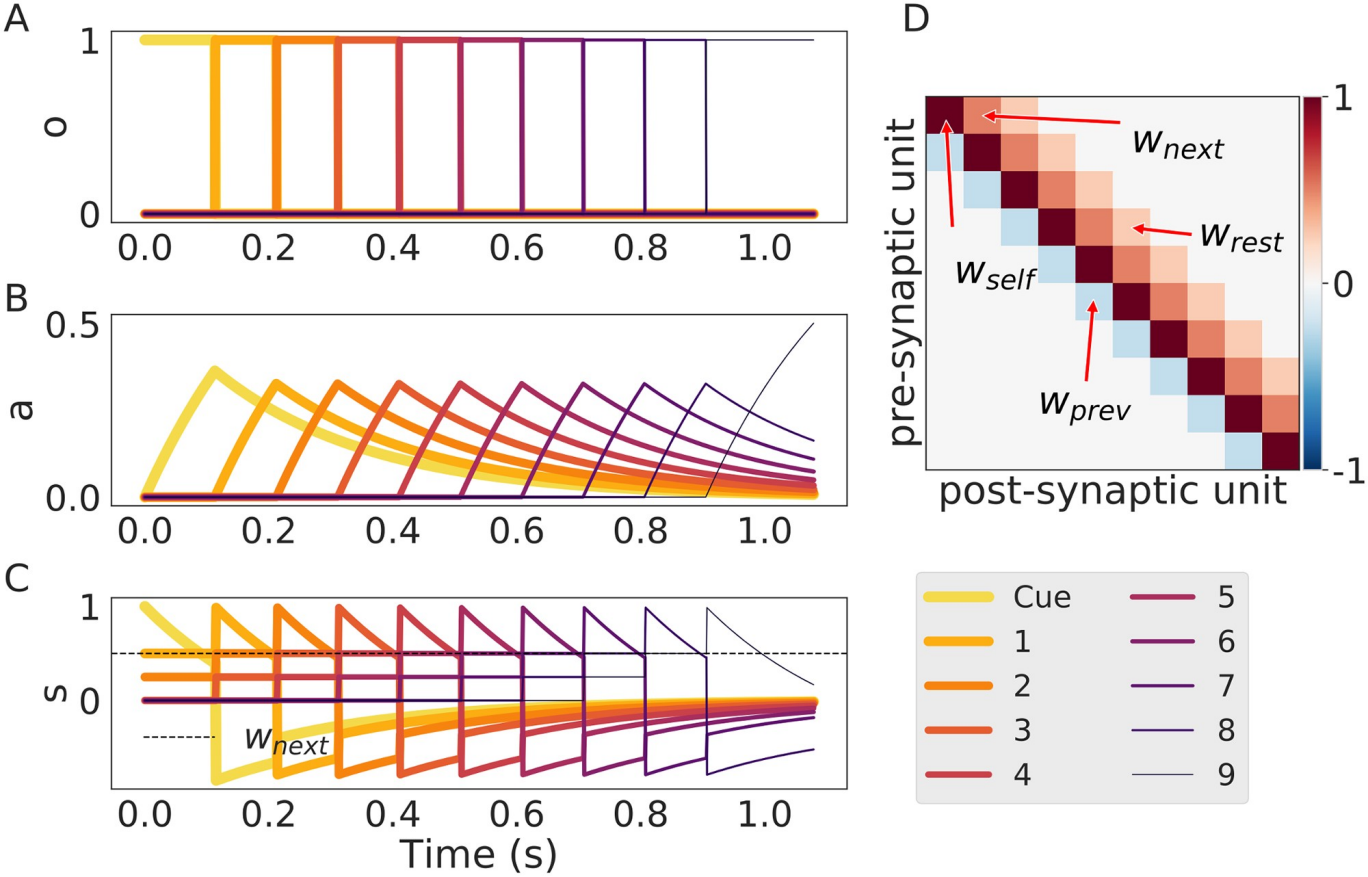
An instance of sequence recall in the model. (A) Sequential activity of units initiated by the cue. (B) The time course of the adaptation current for each unit. (C) The total current *s*, note that this quantity crossing the value of *w*_*next*_*o* (depicted here with a dotted line) marks the transition point from one pattern to the next. (D) The connectivity matrix where we have included pointers to the most important quantities *w*_*self*_ for the self-excitatory weight, *w*_*next*_ for the inhibitory connection to the next element, *w*_*rest*_ for the largest connection in the column after *w*_*next*_ and *w*_*prev*_ for the connection to the last pattern that was active in the sequence.

In Fig 2A we show a case of successful sequential recall in the network with the connectivity matrix depicted in Fig 2D. Here we handcrafted the connectivity matrix to illustrate the unfolding of the following dynamics. Once the first pattern gets activated (*o*_*i*_ = 1) as a result of an external cue (current input *I*(*t*) to all the units belonging to the pattern) the adaptation current *a*_*i*_ depicted in Fig 2B starts growing and, in consequence, the self-excitatory current *s*_*i*_ becomes smaller. At some point, the self-excitatory current *s*_*i*_ is going to become weaker than the feed-forward current *s*_*i*+1_, which the next pattern in the sequence receives. Then, the competitive WTA mechanism mediates the activation of the next pattern (*o*_*i*+1_ = 1) and suppresses the current one (*o*_*i*_) by competition. These dynamics are self-sustained and the cycle repeats until the end of the sequence. We depict the profile of such transitions in Fig 2C. The total time that the pattern stays activated is defined as the persistence time *T*_*per*_ (as used in [53]) and depends on the interplay between the connectivity matrix, the bias term and the adaptation. We present typical values of the network parameters in Table 1.

**Table 1:** Network’s parameters and quantities.

Symbol	Name	Values
*τ*_*s*_	Synaptic time constant	10 *ms*
*τ*_*a*_	Adaptation time constant	250 *ms*
*g*_*a*_	Adaptation gain	0 − 2.5 (units of *w*, control)
$\tau_{z_{pre}}$	Pre synaptic z-filter time constant	5 − 150 *ms*
$\tau_{z_{post}}$	Post synaptic z-filter time constant	5 *ms*
*τ*_*p*_	Probability traces time constant	5 *s*
*σ*	Standard deviation of s values	0 − 3
*T*_*per*_	Persistence time	50 − 3000 *ms* (controlled)
*T*_*p*_	Pulse time	100 *ms*
IPI	Inter pulse interval	0 *ms*
H	Number of hypercolumns	variable
N	Total number of units	variable

### 2.2 Persistence time

Two important characteristics of sequence dynamics are the order in which the patterns are activated (the serial order) and the temporal structure of those activations (the temporal order) [54]. In our model the serial order is determined by the differential connectivity between the units belonging to the currently activated pattern and those of all the other patterns. In general, the next pattern activated will be the one for which the quantity Δ*w*_*next*_ = *w*_*self*_ − *w*_*next*_ is smaller. The persistence time or temporal information of the sequence on the other hand is determined by the interplay between the connectivity of the network and the dynamical parameters of the network. We now proceed to characterize this relationship analytically. From the deterministic trajectories (see S1 Appendix) we can find the time point at which the currents from two subsequent units are equal, *s*_*i*_(*t*) = *s*_*i*+1_(*t*), as this results in the transition and thus determines the persistence time, *T*_*per*_. Solving for *t* we can estimate the persistence time, *T*_*per*_, in terms of the other network parameters:

$$\begin{array}{c} T_{per}=\tau_{a}\log(\frac{1}{1-B})+\tau_{a}\log(\frac{1}{1-\frac{\tau_{s}}{\tau_{a}}}) \end{array}$$ (4)

$$\begin{array}{cc} B & =\frac{w_{self}-w_{next}+\beta_{self}-\beta_{next}}{g_{a}}\\ & =\frac{\Delta w_{next}+\Delta \beta_{next}}{g_{a}} \end{array}$$ (5)

**Fig 3:**
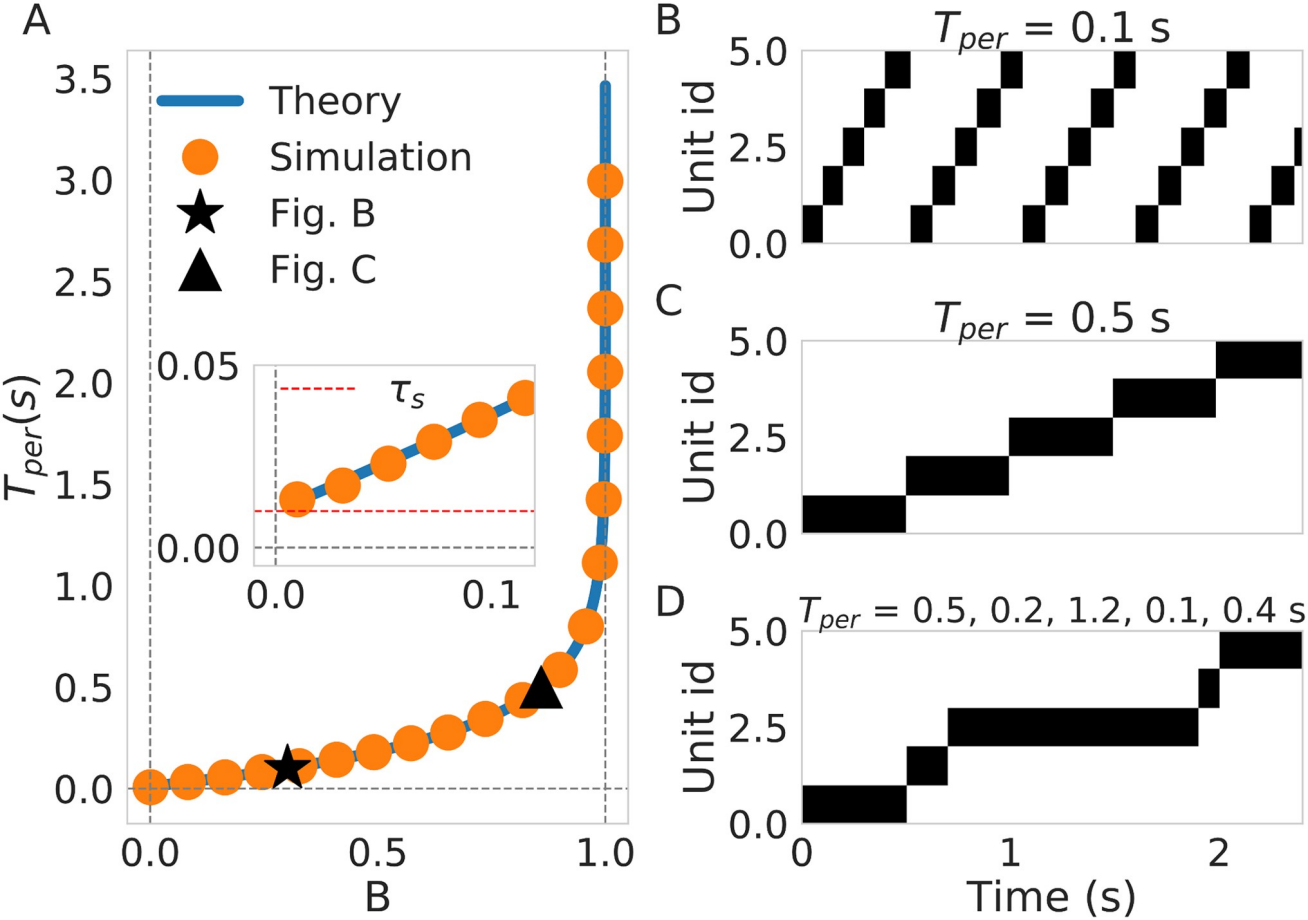
Systematic study of persistence time *T*_*per*_. (A) *T*_*per*_ dependence of B. The blue solid line represents the theoretical prediction described in Eq 4 and the orange bullets are the result of simulations. Inset depicts what happens close to *B* = 0 where we can see that the lower limit is the time constant of the units *τ*_*s*_. (B) An example of sequence recall where *T*_*per*_ = 100 *ms*. This example corresponds to configuration marked the black star in (A). (C) example of sequence recall with *T*_*per*_ = 500 *ms*. This example corresponds to the configuration marked with a black triangle in (A). (D) Recall of a sequence with variable temporal structure (varying *T*_*per*_. The values of *T*_*per*_ are 500, 200, 1200, 100, and 400 ms respectively.

The parameter B in Eq 5 condenses information regarding the connectivity *w*, bias terms *β*, and adaptation strength *g*_*a*_. From Eq 4 we can infer that *T*_*per*_ is defined only for 0 < *B* < 1. This sets the conditions for how the weights, bias and external input interact with the adaptation parameters in order for the sequence to be learned and recalled. The straightforward interpretation for *B* < 1 is that the adaptation has to be strong enough to overcome the effects of the other currents, while *B* > 0 sets the connectivity conditions for sequence recall to occur (*w*_*self*_ > *w*_*next*_). As illustrated in Fig 3A *T*_*per*_ is small for *B* ≈ 0 and diverges to infinity as *B* ≈ 1. This facilitates the interpretation of *B* as a unitless parameter whose natural interpretation is the inverse of transition speed, as shown in the examples provided in Fig 3B and 3C.

Controlling the individual persistence times of different patterns (the temporal structure) through short-term dynamics has been discussed previously in the literature [40]. In our network the temporal structure of the sequence is also controlled by the adaptation dynamics. We illustrate this in Fig 3D where by choosing specific values for the adaptation gain, *g*_*a*_, precise control of the *T*_*per*_ is achieved for every attractor.

For illustration purposes, Eq 4 is given for the case of orthogonal patterns and a single hypercolumn. In the general case with multiple hypercolumns it is possible that not all local transitions within a pattern (in different hypercolumns) occur at the same time. Moreover, as we recall sequences with non-repeating patterns the adaptation effects are not specified. A full treatment, that handles both the modular effects of non-overlapping elements and adaptation effects is provided in the supplementary material (see S1 Appendix).

### 2.3 Learning

So far we have shown that our model can support sequence recall and control of the temporal structure through the adaptation dynamics. We now show that when the network is subject to the right spatio-temporal input structure then associative Hebbian learning is sufficient to induce the learning of the asymmetric connectivity structure characteristic of sequence recall [52]. Based on previous work [39], we use the BCPNN learning rule in its incremental on-line version [55] with learning mediated through asymmetric synaptic time traces. The version of the BCPNN learning rule utilized here is an adaptation of the discrete learning rule (presented in [46]) to a continuous setting.

$$\begin{array}{cc} \tau_{z_{pre}}\frac{dz_{i}}{dt}=o_{i}-z_{i} & \tau_{z_{post}}\frac{dz_{j}}{dt}=o_{j}-z_{j} \end{array}$$ (6)

$$\begin{array}{cc} \tau_{p}\frac{dp_{i}}{dt}=z_{i}-p_{i}\;\;\tau_{p}\frac{dp_{ij}}{dt}=z_{i}z_{j}-p_{ij} & \tau_{p}\frac{dp_{j}}{dt}=z_{j}-p_{j} \end{array}$$ (7)

$$\begin{array}{cc} w_{ij}=\log(\frac{p_{ij}}{p_{i}p_{j}}) & \beta_{j}=\log(p_{j}) \end{array}$$ (8)

**Fig 4:**
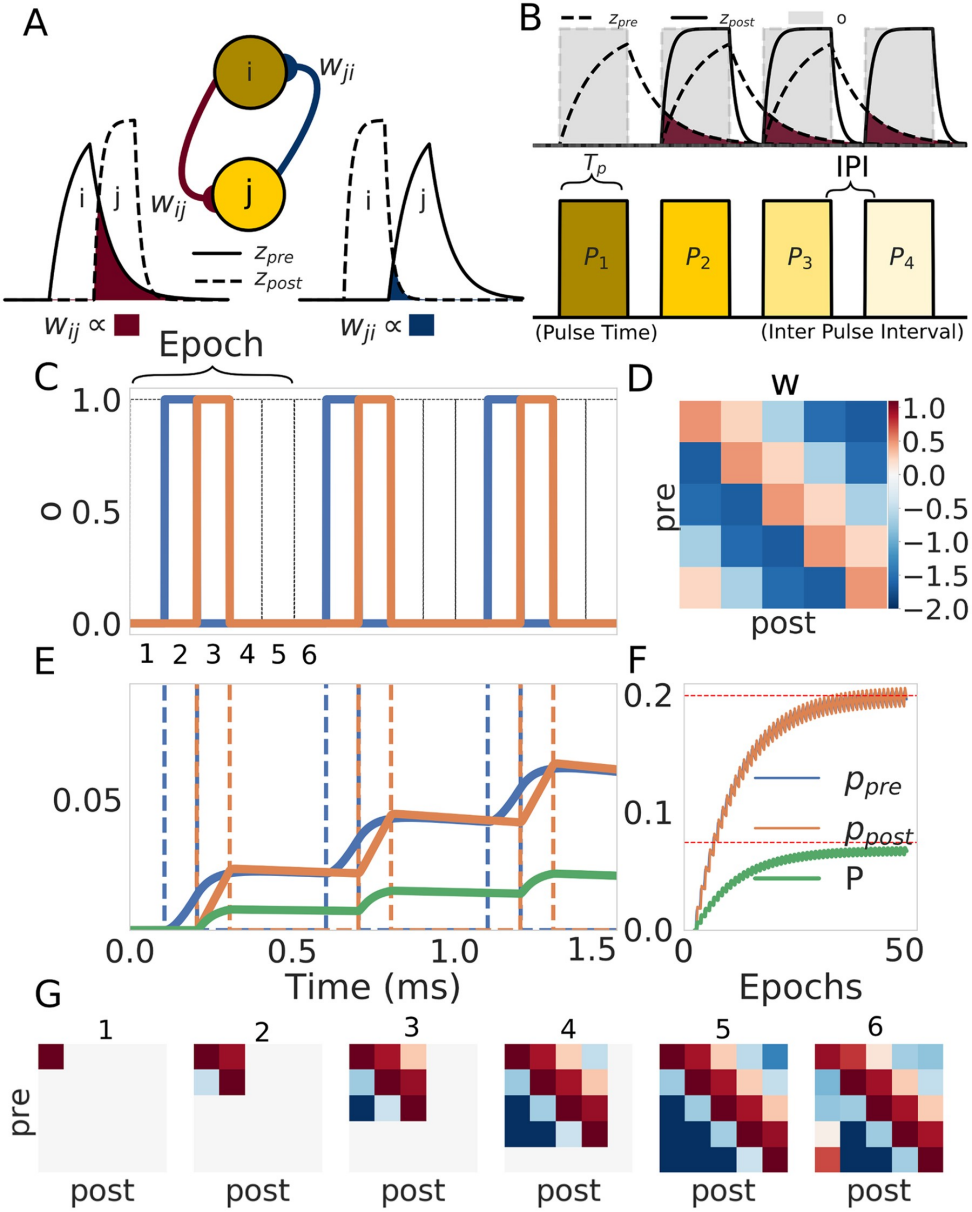
Sequence learning paradigm. (A) Relationship between the connectivity matrix *w* and the z-traces. The weight *w*_*ij*_ from unit *i* to unit *j* is determined by the probability of co-activation of those units which in turn is proportional to the overlap between the z-traces (show in dark red). The symmetric connection *w*_*ij*_ is calculated through the same process but with the traces flipped (here shown in dark blue). Note that the asymmetry of the weights is a direct consequence of the asymmetry of the z-traces. (B) Schematic of the training protocol. In the top we show how the activation of the patterns (in gray) induces the z-traces. In the bottom we show the structure of the training protocol where the pulse time *T*_*p*_ and the inter-pulse interval (IPI) are shown for further reference. (C) We trained a network with only five units in a single hypercolumn for illustration. The first three epochs (50 in total) of the training protocol are shown for reference. The values of the parameters during training were set to *T*_*p*_ = 100 *ms*, *IPI* = 0 *ms*, $\tau_{z_{pre}}=50\;ms$ and $\tau_{z_{post}}=5\;ms$. (D) The matrix at the end of the training (after 50 epochs). (E) Evolution of the probability values during the first three epochs of training. The probability values of the pre (*p*_*i*_), post (*p*_*j*_) and joint probability (*p*_*ij*_) evolve with every presentation. Note that the same color code is used in images C, E and F. (F) Long-term evolution of the probabilities with respect to the number of epochs. The values of the probability traces eventually reach a steady state. (G) Short-term evolution of the weight matrix at the points marked in the first epoch in C. Note that the colors are subjected to the same colorbar reference as in D.

In the spirit of associative learning the BCPNN rule sets positive weights of recurrent connections between units that statistically tend to co-activate and creates inhibitory connections (negative weights) between those that do not. This is reflected in Eq 8, where the connections are determined with a logarithmic ratio between the probability of co-activation (*p*_*ij*_) and the product of the activation probabilities (*p*_*i*_ and *p*_*j*_). Note that if the events are independent the weight between them is zero (*p*_*ij*_ = *p*_*i*_ *p*_*j*_). Nevertheless, basic associative learning can only bind units that are active simultaneously. In order to bind units that are not simultaneously active in time we need an extra mechanism of temporal integration [52]. To overcome this we combine the BCPNN learning rule with the introduction of the z-traces in order to create temporal associations between units that are contiguous in time [56]. The z-traces, defined in Eq 6, which can be thought of as synaptic traces, are a low-passed filtered version of the unit activations *o* and dynamically track the activation as shown in the top of Fig 4B. To approximate the probabilities of activation (*p*_*i*_ and *p*_*j*_) and co-activation (*p*_*ij*_) the z-traces are accumulated over time in agreement with Eq 7, which implements an on-line version of the exponentially weighted moving average (EWMA). As illustrated in Fig 4A, asymmetry in the connectivity matrix arises from having two z-traces, a pre-synaptic trace with a slow time constant $\tau_{z_{pre}}$ and a fast post-synaptic trace with a fast time constant $\tau_{z_{post}}$ [39]. In short, the z-traces work as a temporal proxy for unit activation that allow us to use the probabilistic framework of the BCPNN rule to learn the sequential structure of the input.

The training protocol shown in Fig 4B is driven by the temporal nature of the input and can be characterized by two quantities: the time that the network is exposed to a pattern (this is implemented by clamping the units belonging to the corresponding pattern through *I* in Eq 1) called the pulse time, *T*_*p*_, and the time between the presentations of two patterns referred as the inter-pulse-interval (IPI). In the following we use a homogeneous training protocol where the values of the *T*_*p*_ and IPI are the same for every pattern in the sequence.

The network’s weights were learned using a training protocol where the patterns were presented sequentially for a number of epochs (50 epochs in the example illustrated in Fig 4C–4G). With every presentation of the stimulus the probability traces *p* grow accordingly (see Fig 4E), slowly evolving to their steady state value (Fig 4F). While the steady state weight matrix that results from training reveals asymmetric connectivity (Fig 4D), the sequential structure of the input is learned as early as during the first epoch, as can be observed in Fig 4G. This demonstrates that the sequential structure of the input has been successfully learned by the BCPNN rule with the help of the z-traces.

**Fig 5:**
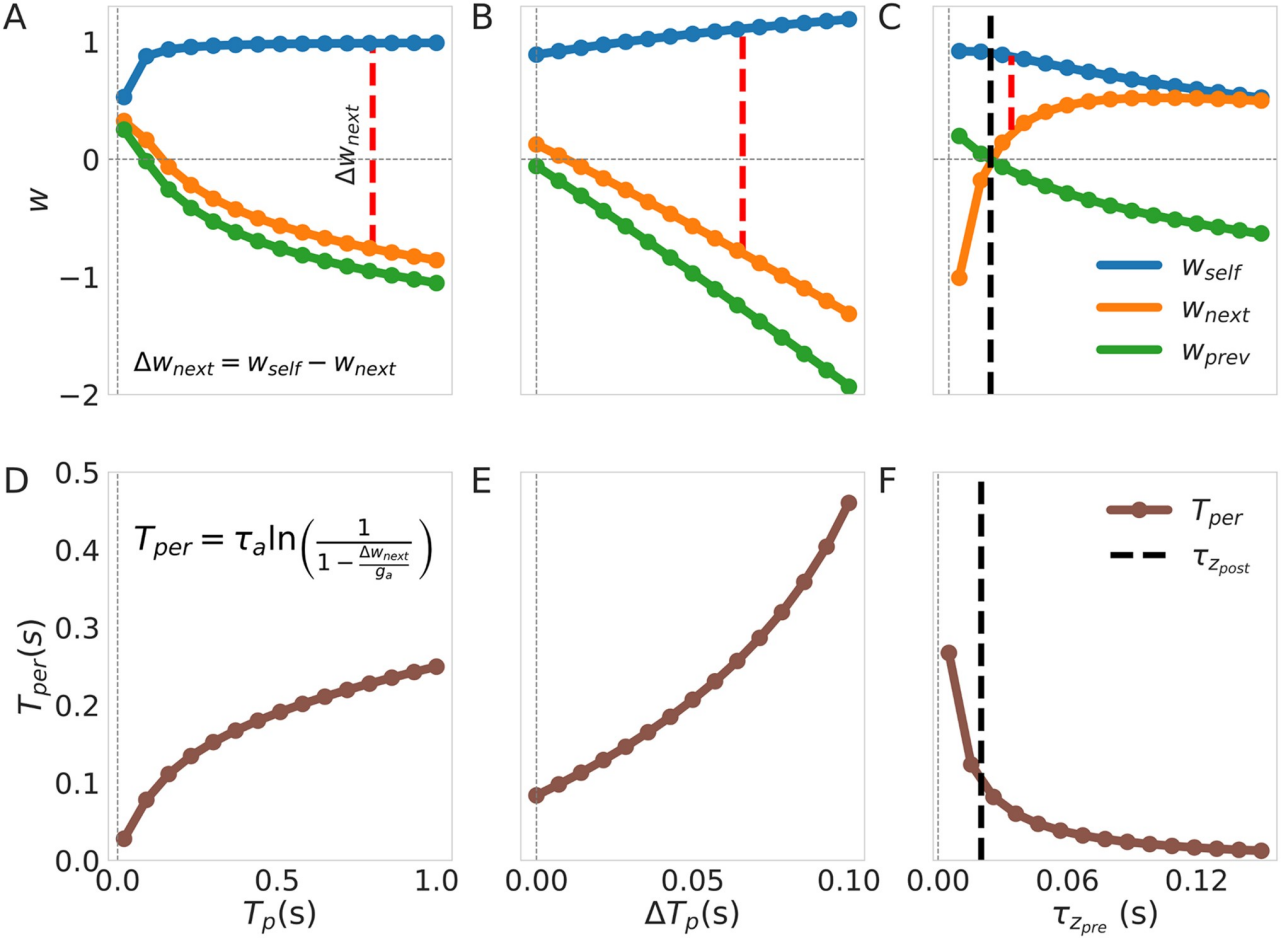
Characterization of the effect of training in the connectivity weights and persistent times. The equation on the inset in D relates *T*_*per*_ to Δ*w*_*next*_ = *w*_*self*_ − *w*_*next*_ which we show as dashed red lines in each of the top figures (note that here Δ*β* = 0 as we trained with an homogeneous protocol). When the parameters themselves are not subjected to variation their values are: *T*_*p*_ = 100 *ms*, *IPI* = 0 *ms*, $\tau_{z_{pre}}=25\;ms$, $\tau_{z_{post}}=20\;ms$ for all the units. (A-C) Show how the weights depend on the training parameters *T*_*p*_, inter pulse interval and $\tau_{z_{pre}}$, respectively, whereas (D-E) illustrate the same effects on *T*_*per*_. Here we are providing the steady state values of *w* obtained after 100 epochs of training.

We characterized the relationship between the connectivity matrix (*w*_*self*_, *w*_*next*_ and *w*_*prev*_) and the training protocol parameters (the pulse time *T*_*P*_, the inter-pulse-interval, IPI, and the two time constants of the synaptic traces $\tau_{z_{pre}}$ and $\tau_{z_{post}}$). We summarize our findings and its relationship to the persistence time, *T*_*per*_, in Fig 5. Larger values of *T*_*p*_ lead first to an increase in the value of *w*_*self*_ followed by its stabilization thereafter and to a decrease in the value of *w*_*next*_ (Fig 5A). This can be explained by the fact that while the ratio between self co-activation and the total training time remains more or less constant (stabilizing *w*_*self*_) the co-activation between units becomes a smaller portion of the whole training protocol effectively reducing the estimating of *p*_*ij*_ (making *w*_*next*_ smaller). In consequence, the rate of *T*_*per*_ growth becomes constant with larger *T*_*p*_ giving a logarithmic encoding of time (Fig 5D). In contrast, larger IPIs lead to monotonic increments and decrements in *w*_*self*_ and *w*_*next*_ respectively (Fig 5B). The reason for this is that larger IPIs bring about an overall longer training protocol and after the co-activation of the units ceases, the product *p*_*i*_*p*_*i*_, decreases faster than *p*_*ii*_ leading to a larger *w*_*self*_. The value of *w*_*next*_, on the other hand, is rendered smaller by larger IPIs as a consequence of the unit’s activations begin further apart in time. It follows that *T*_*per*_ increases faster with larger IPIs as both *w*_*self*_ and *w*_*next*_ separate farther and farther with growing inter pulse intervals (Fig 5E). The effect of the z-filters time constant *τ*_*z*_ in the weights can be described as diminishing the difference between *w*_*self*_ and *w*_*next*_ (Fig 5C). The results can be explained by interpreting the effect of increasing $\tau_{z_{pre}}$ as spreading more and more the activation in time rendering the co-activations less meaningful overall (co-activation probability drops). This results in a diminishing value of *T*_*per*_ as the difference between weights Δ*w*_*next*_ drops with larger values of $\tau_{z_{pre}}$ (Fig 5F). Note here that the point at which $\tau_{z_{pre}}$ becomes larger than $\tau_{z_{post}}$ (marked with a dashed red line) coincides with *w*_*next*_ becoming larger than *w*_*prev*_ as we should expect. The reasoning for *w*_*pre*_ is analogous to that of *w*_*next*_ with the only difference in synaptic time constant ($\tau_{z_{post}}$ instead of $\tau_{z_{pre}}$).

**Fig 6:**
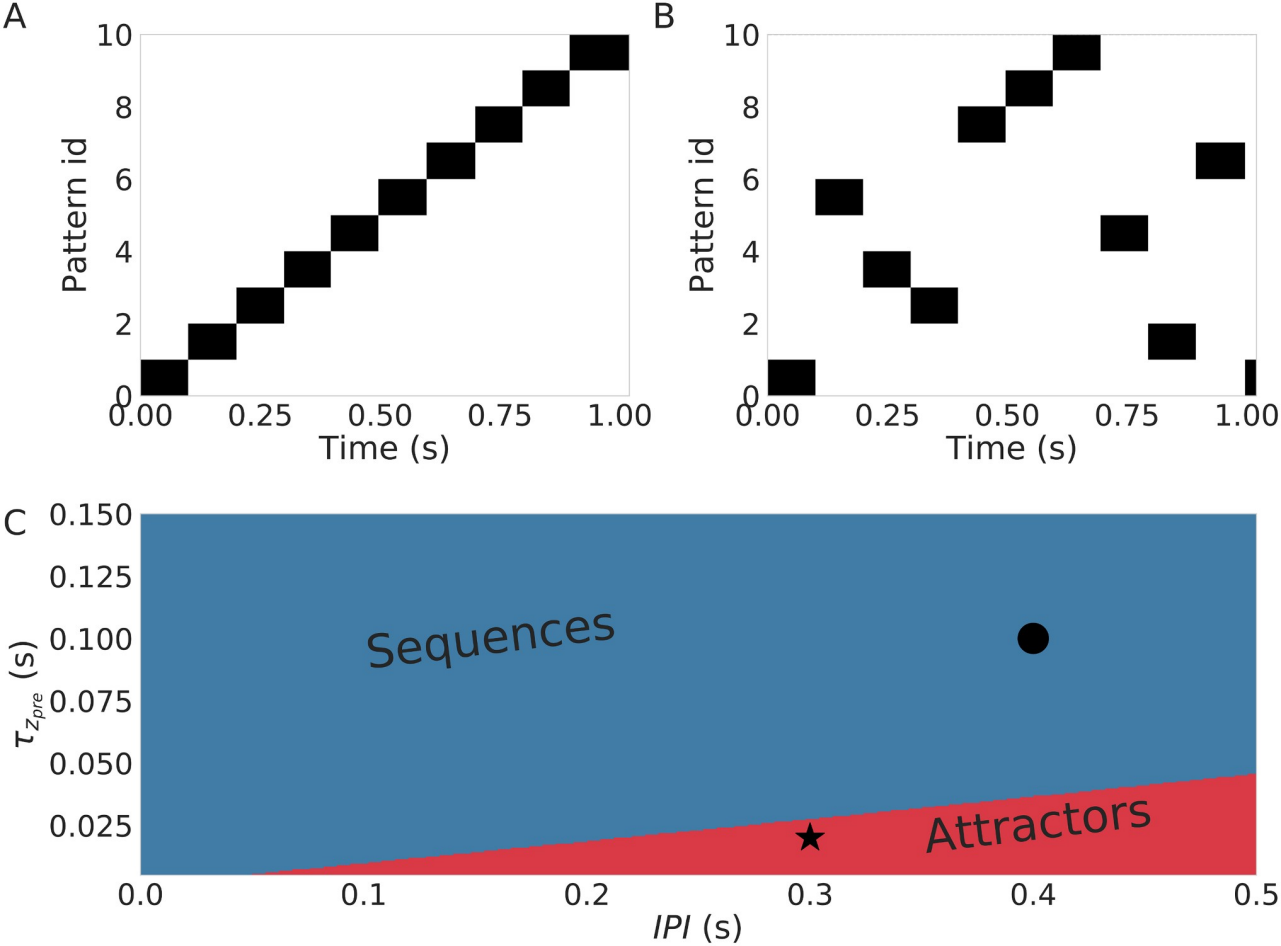
Transition from the sequence regime to a random reactivation regime. (A) An example of a sequential (ordered) activation of patterns. (B) Unordered reactivation of the learned attractors. (C) The two regimes (sequential in blue and random reactivation of attractors in red) in the relevant parameter space spammed by $\tau_{z_{pre}}$ and inter pulse interval. The examples in (A) and (B) correspond to the black dot and the star, respectively.

We have shown so far that the temporal structure of the input determines the temporal structure of the recall (Fig 5D–5F). We now show that the value IPI can change the recall phase from a sequence regime, where the patterns are tied in time, (Fig 6A) to a regime where the attractors are learned but not their temporal arrangement (Fig 6B). In this regime the network undergoes an unordered reactivation of the attractors in the recall phase. In general, to bridge a longer inter-pulse-interval, a longer $\tau_{z_{pre}}$ is required, as illustrated in Fig 6C. The idea is that $\tau_{z_{pre}}$ provides a temporal window of integration withing which patterns can be tied into a sequence. So, the larger the window is, the longer are the IPIs can be to still ensure the sequential memory.

### 2.4 Noise

**Fig 7:**
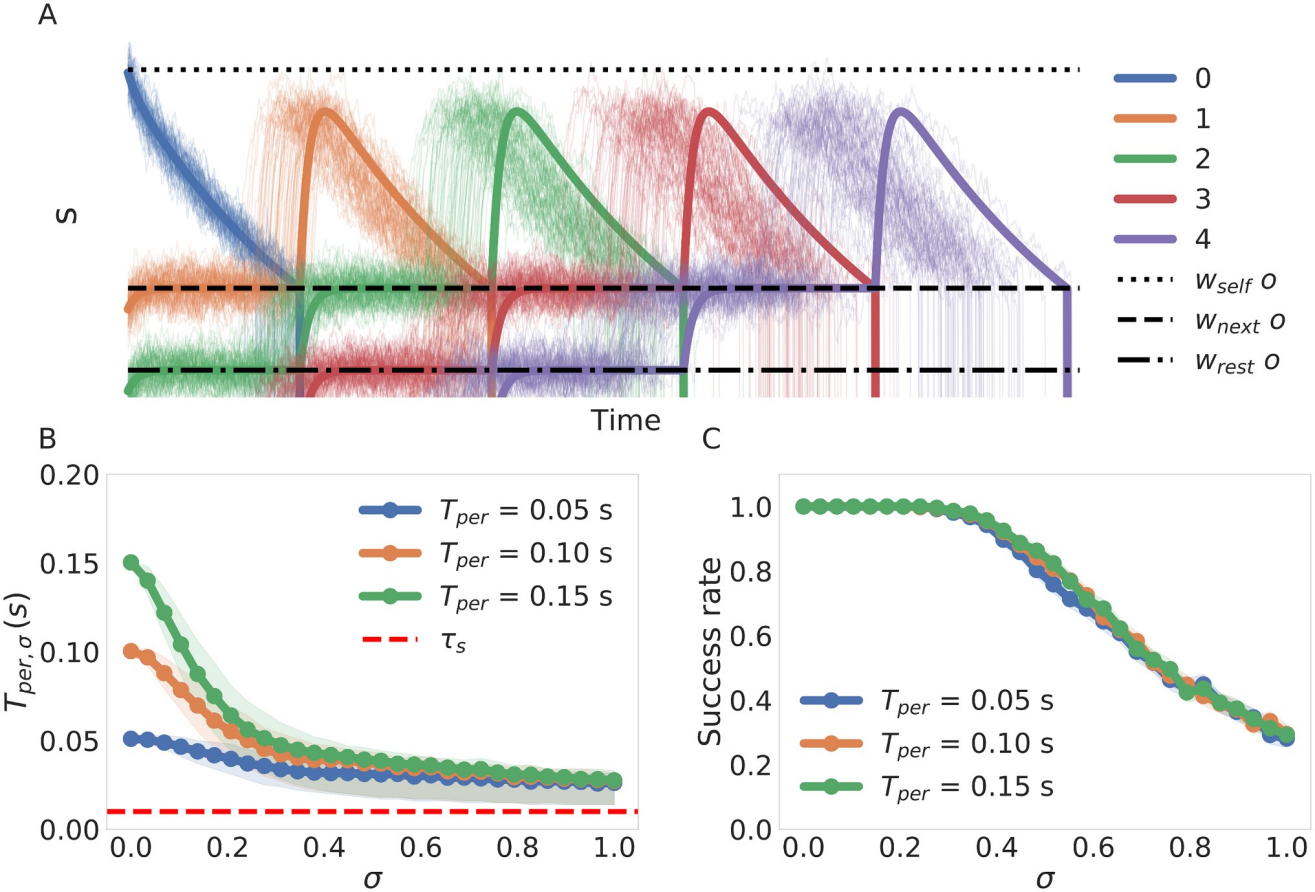
Effects of noise reflected in current trajectories and persistence times. (A) An example of current trajectories subjected to noise. The solid lines indicate the deterministic trajectories the system would follow in the zero noise case. In dotted, jagged and dashed lines we depict the currents induce *w*_*self*_, *w*_*next*_ and *w*_*rest*_ for reference. (B) Change in the average of the actual value of *T*_*per*_ for different levels on noise. We Shaded the area between the 25th and the 75th percentile to convey and idea of the distribution for every value of *σ* (C) Success rate vs noise profile dependence on *T*_*per*_. We ran 1000 simulations of recall and present the ratio of successful recalls as a function of *σ*. Confidence intervals from the binomial distribution are too small to be seen.

We also tested whether sequence recall in the network was robust to noise by controlling the level of noise with the parameter *σ* in Eq 1. Additive noise manifest itself in stochastic trajectories where pattern to pattern transitions happens earlier (Fig 7A). This phenomenon is illustrated clearly with the red and purple lines in Fig 7A where compared to their deterministic counterparts (solid lines) the noisy trajectories (thin lines) make the transition as soon as the variations in *s* drive them under the transition point (*w*_*next*_ *o*). Therefore, the persistence time in a network operating in a noisy regime will be a stochastic variable (denoted *T*_*per*,*σ*_) whose mean will be lower than the persistence time *T*_*per*_ present in the deterministic regime. The mean value of *T*_*per*,*σ*_ decays systematically with increasing *σ* and quickly converges to a common value independent of the value of *T*_*per*_ for the deterministic regime set by controlling *g*_*a*_ (Fig 7B). To examine whether a sequence with lower values of *T*_*per*_ is less likely to be recalled correctly under the influence of noise we cued the sequence 1000 times for every value of *σ* and estimated the success rate by dividing the number of times that the sequence was correctly recalled in its entirety by the number of trials (1000). With this information we constructed the success rate vs noise profile shown in Fig 7C where we can observe that the success rate is identical for different values of *T*_*per*_. We conclude that *T*_*per*_ has no effect on the sensitivity of the recall process is noise. This facilitates the study of the effect of noise as we can disregard variations on *T*_*per*_.

**Fig 8:**
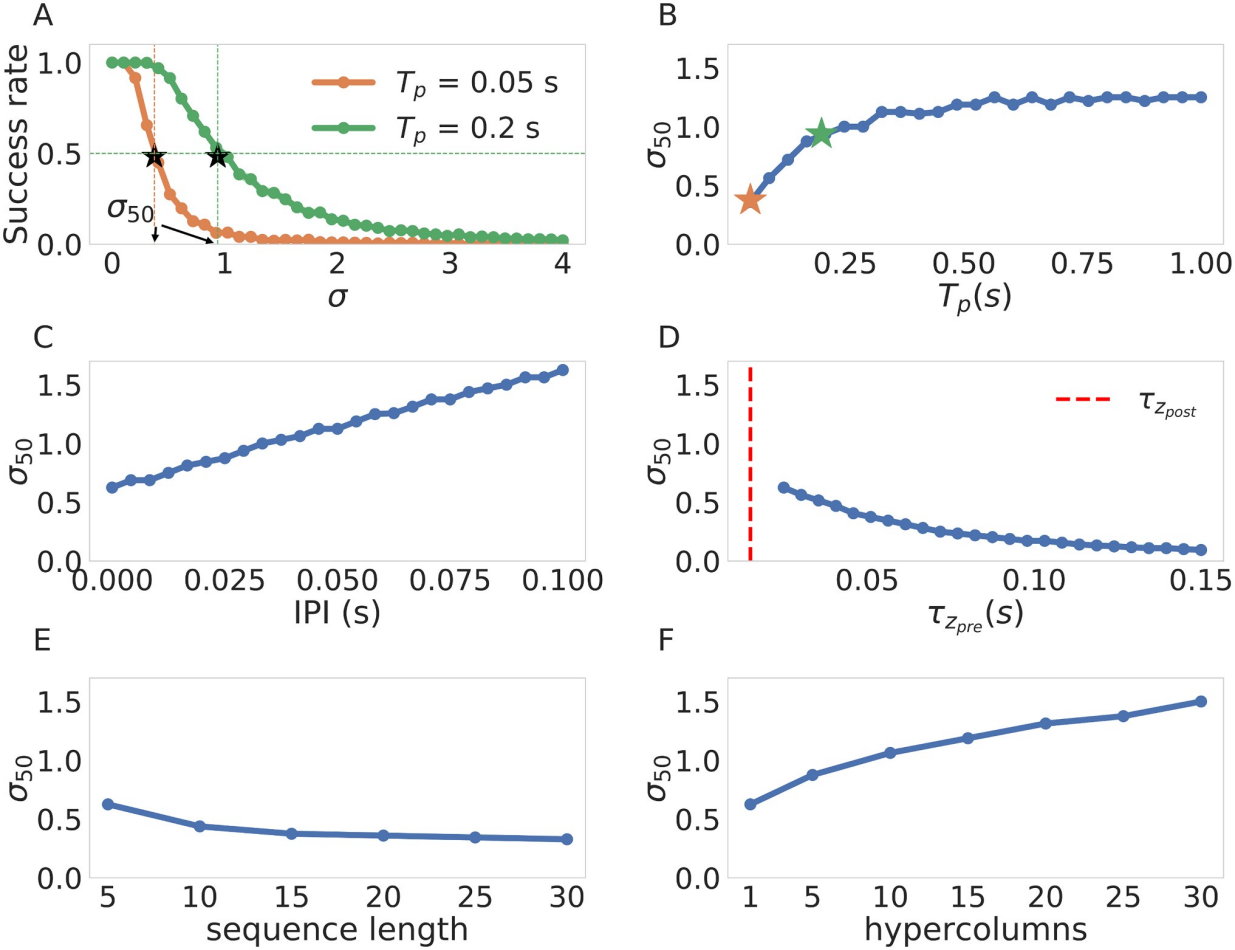
Sensitivity of network performance to noise for different parameters. The base reference values of the parameters of interest are: *T*_*p*_ = 100 *ms*, *IPI* = 0 *ms*, $\tau_{z_{pre}}=25\;ms$, $\tau_{z_{post}}=15\;ms$, sequence length = 5, #hypercolumns = 1. (A) Two examples of the success vs noise profiles (*T*_*p*_ = 50 *ms*, 200 *ms*). The value of *σ*_50_ is indicated in the abscissa for clarity, note that smaller *σ*_50_ implies a network that is more sensitive to noise (the success rate decays faster). (B) *σ*_50_ variation with respect to *T*_*P*_. We also indicate the *σ*_50_ for the values of *T*_*p*_ used in (A) with stars of corresponding colors.(C) *σ*_50_ variation with respect to the inter pulse intervals. (D) *σ*_50_ variation with respect to the value of $\tau_{z_{pre}}$. (E) *σ*_50_ variation with respect to sequence length. (F) *σ*_50_ variation with respect to the number of hypercolumns.

Next we systematically characterized the sensitivity of the network to noise as a function of the training parameters by calculating *σ*_50_ (the value at which the success rate falls below fifty percent, see Methods). We illustrate the nature of *σ*_50_ in Fig 8A, please note that larger *σ*_50_ implies that a system is less sensitive to noise and vice versa. Having estimated *σ*_50_ for different values of *T*_*p*_ we conclude that the network becomes less sensitive to noise with longer values of *T*_*p*_, as shown in Fig 8B. This can be explained by the fact that training with longer pulses increases the distances between the weights (and therefore the distance between the currents), as previously shown in Fig 5A. We can see the same effect by increasing the inter pulse interval in Fig 8C, where the separation of weights produced by larger IPIs leads to a similar outcome. The opposite effect is observed with longer values of $\tau_{z_{pre}}$ where the system becomes more sensitive with longer values of $\tau_{z_{pre}}$, as shown in Fig 8D. We can appeal again to the structure of the weights in Fig 5C to explain these results as an outcome of the weights and therefore the current being less differentiated among themselves leading to failures in sequence recall.

We also report two relevant noise effects not related to the connectivity. First, we show in Fig 8E that the network becomes more sensitive to noise for longer sequences. This can be explained by considering each pattern-to-pattern transition as a possible point of failure. Naturally, adding more links to the chain makes the recall of the sequence more likely to fail at some point (i.e. not recall all patterns in the right order). Finally, in Fig 8F we observe a scaling effect in how robust the network is with the number of hypercolumns. This can be explained using the fact a network with more hypercolumns posses a higher degree of recurrent connectivity. Every time there is a mis-transition in any of the units the recurrent connectivity channels the currents of the units where the transition occurred correctly as an error correction mechanism assuring the successful completion of the sequence more often than not. In a more abstract language the more hypercolumns the network possess, the less likely it is for enough transitions to occur such that the network state is pushed out of the basin of attraction of the next pattern. Therefore, the more hypercolumns the network possess, the more robust it is to noise and hence the observed scaling.

### 2.5 Overlapping representations and sequences

Previous work with attractor models has shown that it is possible to store attractor states with overlapping representations (i.e. patterns that shared a unit activation in some hypercolumns) [55, 57]. We test here whether our network is able to store and recall overlapping patterns successfully when they belong to sequences and are recalled as such. This is desirable to increase the storage capacity of our network and to enrich the combinatorial representations that our network can process.

**Fig 9:**
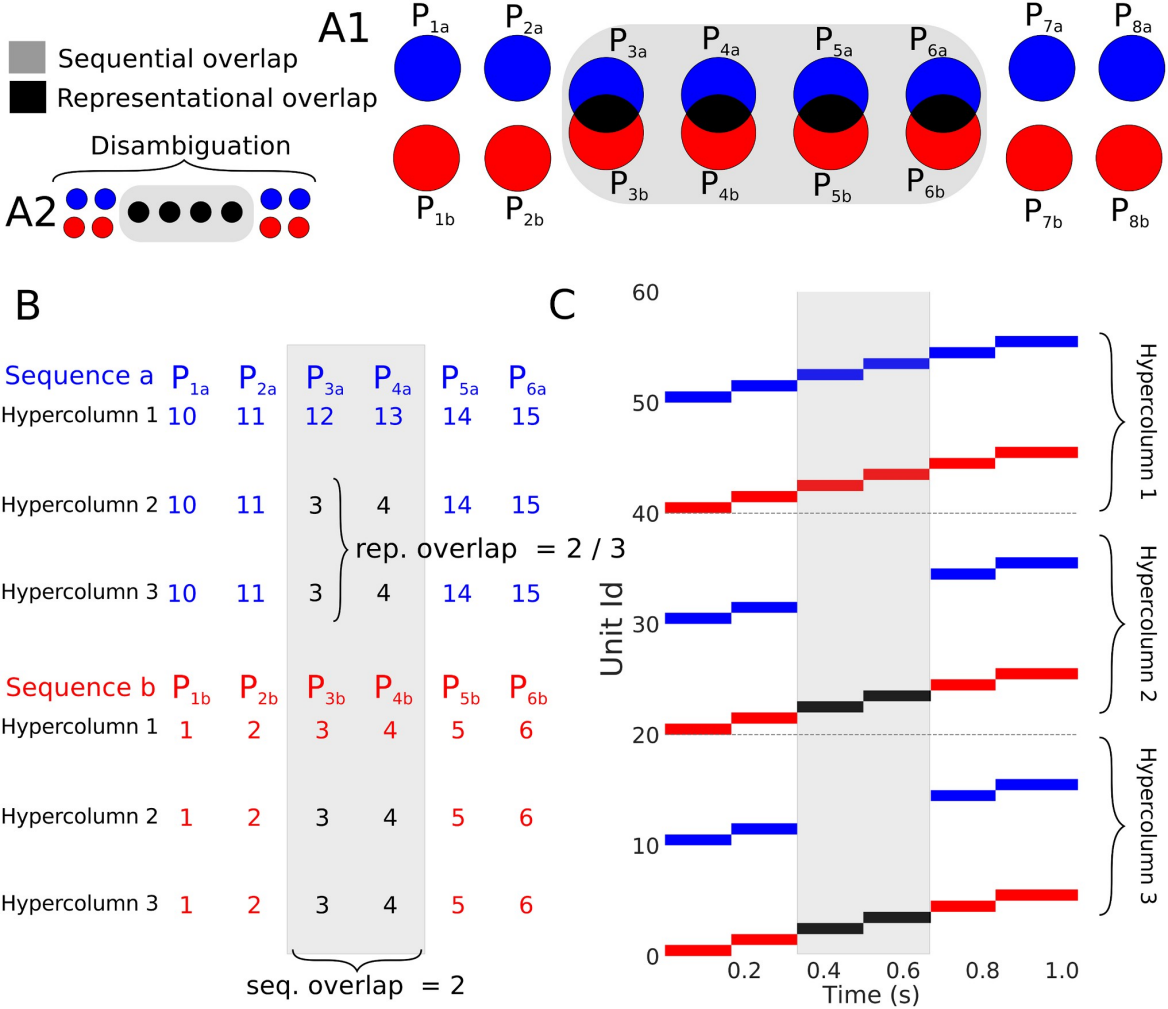
Overlapping representations and sequences. (A1) Schematic of the parameterization framework. Black and gray stand for the representational overlap and the sequential overlap respectively (see text for details) (A2) Schematic of the sequence disambiguation problem. (B) An example of two sequences with overlap. Here each row is a hypercolumn and each column a pattern (patterns *P*_1*x*_, *P*_2*x*_, *P*_3*x*_, *P*_4*x*_, *P*_5*x*_, and *P*_6*x*_). The single entries represent the particular unit that was activated for that hypercolumn and pattern. (C) The superposition of the recall phase for the sequences in (B). Each sequence recall is highlighted by its corresponding color. We can appreciate inside the gray area that the second and third hypercolumns (sequential overlap of 2) have the same units activated (depicted in black). This reflects the fact those patterns have a representational overlap of $\frac{2}{3}$ (two out of three hypercolumns).

Our aim is to characterize the capabilities of our network to store and successfully recall sequences containing patterns with some degree of overlap. As sequences can contain more than a pair of overlapped patterns we propose the following two parameters as a framework to systematically parameterize the problem: 1) the first parameter quantifies the level of overlap between the representation of two patterns and is therefore a spatial measure of overlap, we call this parameter representation overlap. 2) the second parameter is a temporal metric of overlap and quantifies how many patterns between two sequences possess some degree of representational overlap; we call this parameter sequential overlap. A schematic illustration of the general idea is presented in Fig 9A1, where the two parameters, the representational overlap and the sequential overlap, are shown in black and grey, respectively. To be more precise, the representational overlap between two patterns is defined as the proportion / ratio of hypercolumns that share units between the two patterns. We define the sequential overlap between two sequences as the number of patterns in the sequences that possess some degree of overlap (e.g. in Fig 9A1 the sequential overlap is 4). In order to illustrate these concepts we present a detailed example in Fig 9B. The example consists of two six-pattern sequences (i.e. of length six) whose patterns are distributed over three hypercolumns (for example, the first pattern *P*_1*a*_ of sequence a consists in the activation of the unit 10 in each of the three hypercolumns). The two sequences have two pairs of patterns that have some degree of overlap (pairs *P*_3*a*_ − *P*_3*b*_ and *P*_4*a*_ − *P*_4*b*_) and therefore the two sequences have a sequential overlap of 2 as indicated by the gray area in Fig 9B. If we look at patterns *P*_3*a*_ = (12, 3, 3) and *P*_3*b*_ = (3, 3, 3) we can observe that they have the same unit activation in the last two hypercolumns (hypercolumns 2 and 3) and therefore the pair has a representational overlap of $\frac{2}{3}$. The units in the hypercolumns responsible for the representational overlap between the pair are highlighted in black in Fig 9B. Note that the representational overlap is a parameter between 0 and 1, whereas the sequential overlap is an unbounded parameter as sequences can be arbitrarily long.

The limit case when representational overlap is equal to 1 is the domain of sequence disambiguation. We show a schematic of the disambiguation problem in Fig 9A2 where a representational overlap of 1 can be interpreted as the equivalence of both patterns in the sequential overlap section. In this regime the sequential overlap corresponds to the size of the disambiguation window that the network has to bridge to correctly disambiguate the sequence (i.e. ending in *P*_8_ *a* if you started in *P*_1_ *a* in Fig 9A2). Solving sequence disambiguation in the most strict sense requires the network to be able to store the contextual information required to solve correctly the bifurcation at the end of the overlapping section. That is, the network requires to hold the information of what pattern was activated before the disambiguation window for as long as the time it takes for the sequential dynamics to reach forking point.

**Fig 10:**
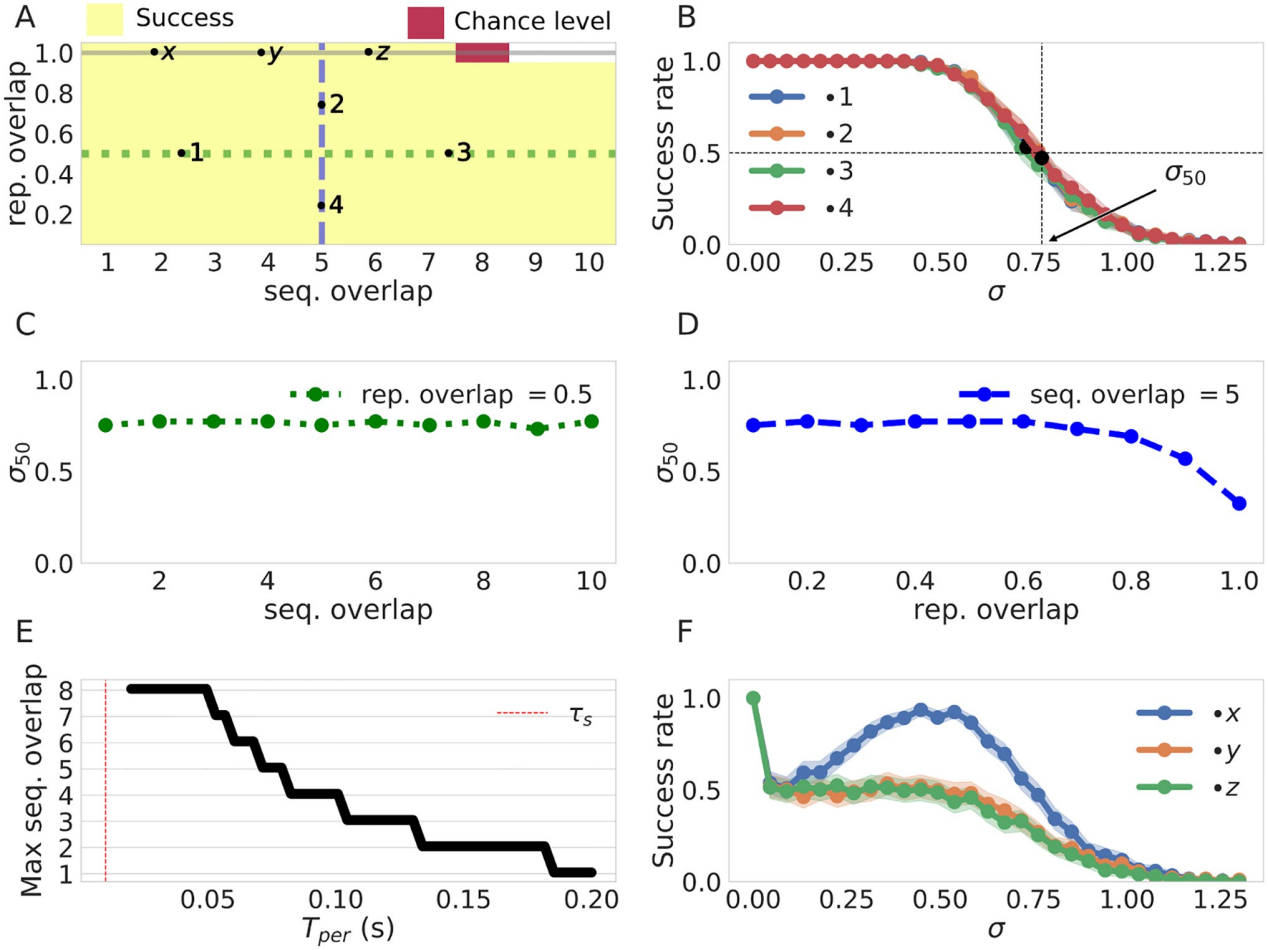
Sequence recall performance for different overlap conditions. The base line values of the parameters of interest are *T*_*p*_ = 100 *ms*, Δ*T*_*p*_ = 0 *ms*, $\tau_{z_{pre}}=25\;ms$, $\tau_{z_{post}}=5\;ms$, sequence length = 10, *H* = 10 and *T*_*per*_ = 50 *ms*. (A) Success rate for pairs of two sequences with different sequential and representation overlaps. We show here the performance over the parameter space. Success here is determined by correct recall of both sequences. Note that the white corner in the top-right is undefined as it corresponds to a degree of sequential overlap that would include either the first or the last pattern in the sequence (B) Success rate vs noise level for the sequences with configurations marked as 1, 2, 3, 4 in A. The values of *σ*_50_ are marked for illustration purposes. (C) *σ*_50_ as a function of the sequential overlap. The values of *σ*_50_ are calculated over the sequences with configurations given in the green horizontal line in A. (D) *σ*_50_ as a function of the representation overlap. The values of *σ*_50_ are calculated over the sequences with configurations given in the blue vertical line in A. (E) max disambiguation as a function of *T*_*per*_. The network loses disambiguation power with long lasting attractors as the memory of the earlier pattern activation reflected in the currents fades. (F) Success rate vs noise profile in the disambiguation regime. The three curves correspond to overlapping sequence configurations marked with x, y, and z in A. Shaded areas correspond to 95% confidence intervals (1000 trials).

In general we should expect that sequences with higher representational and sequential overlaps would be harder to process for the network. To characterize these difficulties systematically we tested for correct sequence recall for sequences in the zero noise condition for all the possible combinations of representation overlap as well as sequential overlap that the network allowed. As can be see in Fig 10A the network can successfully recall overlapping sequences over a wide range of sequential and representational overlaps. The exception to this is the disambiguation regime in top of Fig 10A where we see a failure to recall both sequences when overlapped patterns are identical. Next we studied the recall of sequences with overlapping patterns in the presence of noise. First, we examined the dependence of the success rate on the noise level for a wide array of sequential and representational overlaps (1, 2, 3 and 4 in Fig 10A). The results, as shown by the curves in Fig 10B, illustrate that the success rate vs noise profiles are very similar despite different degrees of sequential and representational overlap. Second, for a fix value of representational overlap (0.5), we calculated *σ*_50_ for all the possible values of sequential overlap (green horizontal line in Fig 10A). We also calculated the values of *σ*_50_ for a fix value of sequential overlap (5) and all the possible values of representational overlap (blue vertical line in Fig 10A). The results (Fig 10C and 10D) show that the network is robust to noise across the spectrum of possible overlaps except when we get close to the sequence disambiguation regime (right part of Fig 10D), where the network becomes more sensitive. Those results together suggests that our neural network can consistently recall sequences correctly over a broad set of overlap conditions.

In the disambiguation regime with no noise (gray line in Fig 10A) the network is able to solve the disambiguation problem successfully up to disambiguation windows of size 8. The disambiguation capabilities of the network are due to memory effects on the dynamics (here capacitance effects mediated by *τ*_*s*_). In fact, we show in Fig 10E that the longer the persistence times (and therefore the more time for the memory to fade) the smaller is the disambiguation window that the system can resolve. Contrary to the results above the network is brittle in the sequence disambiguation regime. In particular, the success rate decays extremely fast in the presence of noise as show in Fig 10F. However, an interesting resonance phenomena occurs for low sequential overlaps (blue curve) where the success rate actually increases with noise. This can be explained with the fact that the noise effectively reduces the mean persistence time *T*_*per*,*σ*_ (as shown before in Fig 7B) which leads to the increased disambiguation power (c.f. 9E). In other words, by reducing the attractors life-time with noise, the network is able to leverage the short-lived information provided by the synaptic traces to successfully perform disambiguation.

## 3 Discussion

We have evaluated a Hebbian-like BCPNN learning rule with asymmetrical temporal synaptic traces as a sufficient principle underlying robust sequence learning in an attractor neural network model. The results have revealed the potential of the network to successfully encode and reliably recall multiple overlapping sequential representations even in the presence of noise. In this context, we have systematically studied the effect of network modularity as well as the role of key temporal parameters of the synaptic learning rule. We have also stressed that our network has the capability to control the temporal structure of the sequential pattern recall by means of an intrinsic adaptation mechanism.

Overall we have found that for a wide range of parameters the network learns sequences with no requirement of fine-tuning (see Fig 5). There exist two regimes where the network fails to learn sequences: 1) when the value of $\tau_{z_{pre}}$ is too small compared to the inter-pulse-interval (IPI) which is the case when the attractors are learned but not linked in time (see Fig 6B) and 2) when the value of $\tau_{z_{pre}}$ is so large that the structure of the network gets diluted as the weights connecting a pattern to its successor become larger than the self-excitatory weights (*w*_*self*_ < *w*_*next*_). The other parameters just modulate this process. This fact coupled with a graceful degradation of the network performance with noise (c.f. Fig 8) shows that the sequence learning capabilities of our network are robust to learning and require no fine tuning of the parameters involved.

### 3.1 Comparison with similar models

**Fig 11:**
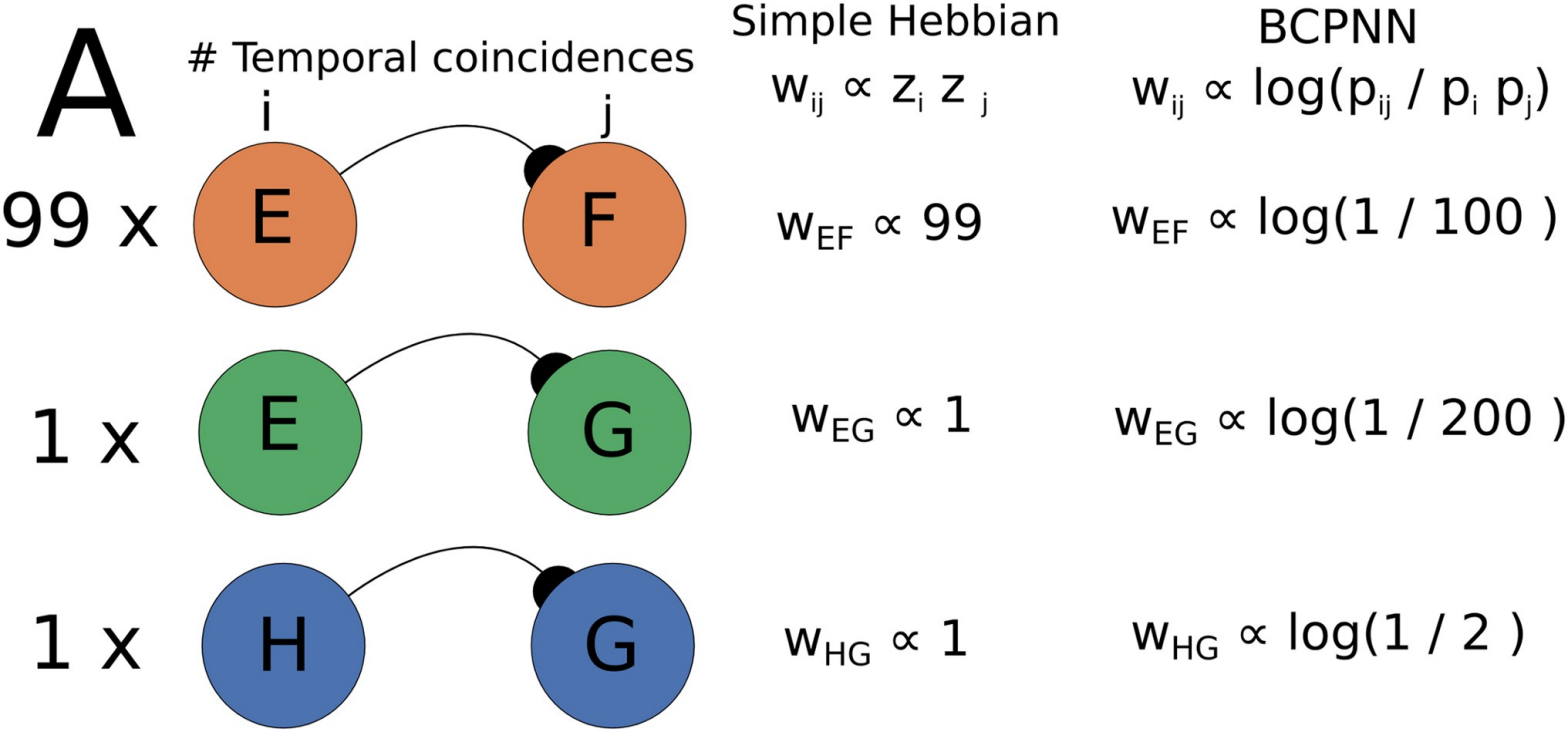
The BCPNN weights temporal co-activations against overall activations. The significance of temporal associations. (A) Here we compare naive simple Hebbian learning with the BCPNN in terms of relative weighting of different temporal associations. In the presented example there are three associations *E* → *F*, *E* → *G*, and *H* → *G* that have been observed 99, 1, 1 occasions respectively. Simple Hebbian learning weights just the frequency of the associations and, as a consequence, *E* → *G* and *H* → *G* end up with the same association weight. The BCPNN, on the other hand, differentiates the weights as it takes into account the total activation probability of each unit, rendering the temporal association *H* → *G* more significant than *E* → *G*.

Previous models have also utilized some of the key components of our model such as the use of temporal traces for hetero-association, competition and the use of adaptation or facilitation to ensure pattern transition [40, 44, 58]. While some of such models provide a study of individual aspects of sequence learning such as the control and characterization of persistence time [40, 44], the analysis of sequence recall under noise [40], or the storage and recall of sequences with some degree of overlap [58], to the best of our knowledge, our approach represents the first systematic treatment of all the aforementioned phenomena under the same framework. In particular, we find that the problem of learning multiple sequences has received scant attention so far. A naive implementation of asymmetric Hebbian learning leads to weights that do not reflect the adequate transition statistics (in the Markov chain sense) of the patterns present during training (see Fig 11A). The BCPNN learning rule that we employ in this work learns the transition statistics by keeping a history of the overall pattern activity in the form of p-traces (see S2 Appendix). It is important to state that most sequence learning models do not implement a naive version of Hebbian plasticity but enhance their plasticity rules with competition motifs (competition among the weights) such as LTD or diverse forms of heterosynaptic plasticity to introduce competition and enhance robustness [41, 42, 58]. However, it is not clear how such competition mechanisms can be balanced to learn temporal associations between patterns that occur with varying frequencies due to their participation in multiple sequences. Such balance that account for this heterogeneous distribution of pattern activation probabilities is offered by the BCPNN as units are automatically connected accordingly to their activation probability history (see Eq 8).

### 3.2 Previous work and biological context

Here we have followed the modelling philosophy aimed at distilling the architecture of the network to its essential characteristics that support and control the phenomenon of interest (sequence learning). In the previous models of particular relevance to our work, complex spike based dynamics and rich biological detail were promoted to provide insights into the biophysical underpinnings of sequence learning in the cortex [39] and as a model of memory consolidation [59]. While the aforementioned contributions provide a more direct mapping between biology and the network, our approach, which reduces the network to its essential characteristics, necessarily dilutes that mapping. Nevertheless, some key design principles emerging from biology are preserved. Below we discuss in more detail the main aspects of the relationship between the dynamical as well as structural properties in our network and the biological substrate that inspired them in the first place.

Local competition, often mediated by lateral inhibition and operating as a normalization mechanism [60], is one of the canonical computational motifs in cortex [47]. In our network competition is modelled locally within each hypercolumn with a hard-wired WTA mechanism, which is not a biologically plausible solution. Douglas and Martin [47] suggested that such a competition mechanism could be implemented by basket or chandelier cells. In the spiking counterparts of our attractor neural network model [39, 59, 61], this computational principle was implemented by means of fast inhibitory basket cells with fixed connectivity and produced compatible outcomes. It is important to point out that the idea of using diverse forms of local competition to achieve pattern selection in sequence recall has been examined previously and extensively in the sequence learning literature [43, 44, 62].

Asymmetric temporal traces have been proven successful to achieve the effect of sequence learning [37, 38, 40, 41, 63, 64]. In our model we have utilized the temporal asymmetric z-traces as the basis of probabilistic learning with the BCPNN learning rule. The degree of asymmetry of the z-traces and its effects on the connectivity matrix have been studied through variations in $\tau_{z_{pre}}$ (Fig 5C). In this framework lower values of $\tau_{z_{pre}}$ would correspond to fast AMPA dynamics [65] while longer values of $\tau_{z_{pre}}$ would correspond in turn to slower NMDA dynamics [66]. Consistently with these observations, throughout this work we have restricted the values of $\tau_{z_{pre}}$ to the 5 − 150 *ms* range. A biological account of the z-traces and their connection to the biochemical cascades that underlie synaptic learning have been presented in a more detailed way by [56].

It is important to point out that synaptic connections learned in our network with the BCPNN learning rule violate Dale’s law, i.e. projections emanating from the same unit can mediate both excitatory and inhibitory effects on the target units. To address this issue, we propose a different interpretation for positive and negative synaptic weights. In the former, they can be straightforwardly interpreted as the conductance between two units, whereas in the latter case we interpret them as a disynaptic connection through an inhibitory interneuron. The argument for the biological plausibility of this arrangement using double bouquet cells as the inhibitory interneurons in this architecture is developed further by [67].

### 3.3 Control of the temporal structure of the sequence

We have shown that the persistence time, *T*_*per*_, of our attractors can be quite effectively controlled through the use of the adaptation gain *g*_*a*_ and less effectively by means of the adaptation time constant *τ*_*a*_ (see Fig 3 and Eq 4). The range of *T*_*per*_ values for the attractor patterns in our network model is within the 10 *ms* and 3.5 *s* range. This in turn means that the duration of our sequences corresponds to the milliseconds to minutes interval (considering sequential lengths of 10 to 100). This range of values is consistent with the variation in sequence duration that [68] found for biological sequences in the hippocampus. While the mechanisms for temporal phenomena under the millisecond scale (inter-aural-scale, [69]) and over the minute scale (circadian rhythms, [70]) are already well understood, the nature and origin of temporal phenomena at the intermediate time scales is still a matter of debate [71]. We believe our work contributes to this debate by offering an intrinsic model of time [72] capable of both, using the taxonomy of [71], the production and reproduction of temporal patterns within the discussed range.

Similarly to previous work [40] we found a logarithmic relationship (Eq 4) between the persistence time, *T*_*per*_, and the network parameters. In their model, Veliz-Cuba et al. (2015) [40] find that by training the network with the right combination of parameters (such as time constants and maximum facilitation), the precise timing of different patterns can be exactly replicated. In our model, we are able to reproduce this effect with only one parameter *g*_*a*_ for the case of orthogonal patterns (see Fig 3D). The case with patterns that share some overlap is more complicated, as it requires adjusting the adaptation gains, *g*_*a*_, more selectively to preserve the duration of all the patterns that contain those units. As far as we know, a firing rate model that is able to adjust its parameters automatically during learning with unsupervised local learning (instead of fixing it by hand) is yet to be found in the literature and remains a matter of future work.

In the work of Murray et al. [44] the control of the temporal structure (*T*_*per*_) is accomplished by means of input from an external network. Although the ability of our network to control the temporal structure rests on internal mechanisms, we could also exploit external input for this purpose. By adding external input to our differential equation during recall and solving the resulting expression (see S1 Appendix) we obtain an expression for the parameter *B* in the following form *B* = (Δ*w*_*next*_ + Δ*β*_*next*_ + Δ*I*(*t*))(*g*_*a*_)^−1^ where Δ*I*(*t*) = *I*_*self*_(*t*) − *I*_*next*_(*t*) is the differential input between the consecutive units in the sequence. By controlling this differential input, the persistence time of attractor states in a given sequence can be modulated. This could be used to build a framework where a generalist network learns the sequential structure of the input and a specialized control network adjusts the temporal structure of the sequence recall suitable for the task at hand.

### 3.4 Sequence disambiguation and overlapping representations

Sequence disambiguation or using past context to determine the trajectory of a sequence has been deemed one of the most important problems that a sequence prediction network should solve [73]. While some networks [74–76] have addressed the problem in their generality, their reliance on supervised learning and lack of biological plausibility remain a matter of concern. There have been a few attempts at the problem of sequence disambiguation in the attractor network framework but most of them rely on non-local learning rules or require an infeasible large number of parameters [52, 77, 78]. Minai et al. [79] proposed an alternative approach using the activity in a random network (what now is called a reservoir) as a source of context information for disambiguation. In their network, activity in the reservoir evolved in a path-dependent way, and inter-network connectivity between the disambiguation network and the reservoir conveyed the necessary information from the latter to the former thus allowing for successful disambiguation. While effective, such networks require another complete layer to keep a dynamical memory, an approach judged to be inefficient. To address this issue, context codes with less overhead have been proposed where, instead of a network, the state of a unit or a collection of units is determined by the dynamical history of the system and that state is then used for disambiguation [80, 81]. In our network, disambiguation can be achieved by building cell assemblies containing a subset of units that are preferentially connected to the subsequent assembly in the sequence. By preferential connectivity we mean that those units posses strong excitatory connections to the units of the subsequent pattern and strong inhibitory connections to the rest. To put it more concretely, the BCPNN learning rule, following its probabilistic nature, will ensure that the non-overlapping parts in a sequence are connected in such fashion by creating excitatory connections between the units in the non-overlapping parts and the subsequent units in the sequence (as they are the only ones that actually appeared together) and strong inhibitory connections between the non-overlapping units and all the units belonging to any other pattern (as they never appeared together). In virtue of the aforementioned connectivity, activation of the units in the non-overlapping part of the assembly (context units) guarantees a transition to the subsequent (correct) pattern. As shown in Fig 10D, the proposed mechanism is very robust to the size of the cell assembly that gets connected preferentially (the non-overlapping part); degradation of the performance under noise only becomes evident when the size of the context code becomes less than 20% of the cell assembly. This is consistent with some experimental evidence of neurons in the hippocampus that fire in such a trajectory dependent fashion [82].

Even in the absence of context units, i.e. with fully overlapping (the same) assemblies in competing sequences, our network can still solve a disambiguation task for sequences sharing two consecutive states in their trajectories (see the resonance phenomena in Fig 10F). While this phenomena allows the network to statistically solve sequence disambiguation for disambiguation windows of size 2, it does not generalize for longer sequential overlaps. One way to handle the problem in a more robust, consistent and transparent fashion is to use a mechanism that preserves the network’s dynamical history in a dynamical variable. In our future work we intend to add such mechanism to the network in the form of currents dependent on the z-traces that facilitate the longer maintenance of the information about past activations and thus support the disambiguation of sequences with more challenging overlaps.

### 3.5 Learning rule stability, competition and homeostasis

The stability of the learning dynamics of a firing rate network subject to associative learning tends to be accomplished by introducing weight dependent terms into weight updates [83]. This constrain is usually motivated and biologically interpreted as a homeostatic mechanism. Sequence learning models are not exempt from this necessity. One of the simpler approaches amounts to combining STDP with hetero-synaptic plasticity [42]. However, it is not straightforward how these two forces should be balanced. There are a plethora of models that rely on weight clipping with arbitrarily handpicked upper and lower limits [40, 44, 62]. While this approach is analytically transparent, fine tuning between potentiation and depression is usually required. In a similar vein, Byrnes et al. [43] introduced a combination of subtractive and multiplicative normalization as a mechanism of weight stabilization, which also has to be arbitrarily tuned. Verduzco-Flores et al. [58] proposed a more complex approach that combines hetero-synaptic competition with a mechanism that limits both the total value of the weights and the total incoming current to a unit in order to achieve stability [41], on the other hand, resorted to a combination of synaptic normalization and multiplicative homoeostasis to avoid runaway excitation. While these two learning rules are able to prevent runaway instabilities and have varying degrees of biological plausibility, the number of parameters involved, and the complexity of the model are excessively high. As opposed to this complexity, the probabilistic nature of our BCPNN learning rule automatically accounts for weight competition during learning leading the network to a stable regime of sequential or attractor dynamics without requiring extra parameters or balancing different forces (as discussed more thoroughly elsewhere [56]).

### 3.6 Limitations and further work

Although multiple studies of the cortical micro-circuitry have revealed distance dependent connectivity profiles [84, 85], we have ignored this design principle in our model. Previous spiking implementations of this model architecture have included to some degree both distance dependent effects in connectivity and distance dependent delays [39, 59, 61], which had impact on the network’s temporal dynamics. In our non-spiking network model the expected implications of such spatio-temporal diversity would be prolonged (temporally spread) attractor reactivation and transition processes. Still there should be no qualitative functional changes in the network’s behaviour as the key mechanisms would not be compromised (although see [86] for a sequence production mechanism that arises itself from asymmetries in the spatial profile of connectivity). Due to the mesoscale nature of our model and interest in network phenomena, we obviously do not account for any dendritic related phenomena in sequence processing such as as the capacity of single neurons to work as sequence recognition devices through spatial effects [87] and the use of distal dendritic inputs to prime sequential activations [88].

In the presented work there are some phenomena that we have not systematically characterized in their generality. For example, in most simulations we exploited temporally homogeneous training protocols. To test the performance of our network under the conditions of varying pulse time, *T*_*p*_, and inter-pulse-interval, Δ*T*_*p*_, across patterns, we have ran preliminary tests and obtained promising results. We intend to conduct a more comprehensive characterization of the network’s behaviour subject to highly variable training protocols (temporal pattern heterogeneity) in our future work.

## 4 Methods

### 4.1 Training and recall protocol

For our training protocol we created a time series **s**(*t*) to represent the input. **s**(*t*) encodes the information about *T*_*p*_ and IPI (Fig 4B). We then performed off-line batch learning of the parameters using the integral formulation of the dynamic equations presented above (Eqs 6 and 7).

To avoid the ill-defined case for *p* = 0 we set the lower bound of *ϵ* = 10^−^7 for the argument of the logarithm. That is, if the value of *p* is less than *ϵ* we equate it to *ϵ*.

For training the two sequences with the overlapping representations we created the sequences in succession but separated among them by 1*s*. This ensured that the sequences in the training protocol were uncoupled from each other.

We consider a pattern to be active if the corresponding units are active for longer than *τ*_*s*_ (the smallest time constant in the system). The sequence is considered to be correctly recalled if by activating the first pattern all the others patterns in the sequence are subsequently activated in that given order. Given that for many possible tasks it suffices that the network state ends in the correct pattern or that only a part of the sequence is recalled correctly our success criteria is rather conservative.

### 4.2 Control and estimation of persistence time

In order to estimate the *T*_*per*_ for a pattern *P* during recall we calculated the difference between the time *t*_1_ at which pattern *P* was activated and the time at which the next pattern was activated *t*_2_. *T*_*per*_ = *t*_2_ − *t*_1_.

As shown in Eq 4, *T*_*per*_ depends on both the weight and bias differences, Δ*w*_*next*_ = *w*_*self*_ − *w*_*next*_ and Δ*β* = *β*_*self*_ − *β*_*next*_, respectively, and the adaptation gain *g*_*a*_. This offers flexibility in controlling the duration of patterns activations by adjusting the adaptation gain *g*_*a*_ as follows: $g_{a}=(\Delta w_{next}+\Delta \beta )(1-\frac{\tau_{s}}{\tau_{a}})(1-\frac{\tau_{s}}{\tau_{a}}-e^{\frac{T_{per}}{\tau_{a}}}{)}^{-1}$. We use this adjustment to control *T*_*per*_ during recall in order to decouple the effects of training from the recall process.

### 4.3 Noise

Noise was included in our simulations as additive white noise with variance $\sigma_{in}^{2}$ in the differential equation for the *s* variable. The current *s*, however, behaves almost as an Ornstein–Uhlenbeck (OU) process and therefore its standard deviation is given by $\sigma_{out}^{2}=\frac{\tau_{s}}{2}\sigma_{in}^{2}$. Based on this fact we characterized the effects of noise with the size of *σ*_*out*_ instead of *σ*_*in*_ The rational behind this choice is that *σ*_*out*_ will be closer to the standard deviation of the variable *s* in Eq 1 and therefore comparable in magnitude to the value of currents in the network. It is important to say that thanks to the separation of times scales (*τ*_*s*_ ≪ *τ*_*a*_) the dynamics of *s* behaves mostly as an OU process and it is only the WTA mechanism around the transition points that induces deviations.

The incorporation of noise to the network makes the trajectories and, thereby, the recall process stochastic. To quantify the recall performance under noise (probability of successful recall at a given level of noise) we averaged the number of correct recalls in a given number of trials. The estimated probability of successful recall $\hat{p}$ follows from a Bernoulli process and we can therefore quantify the uncertainty of our estimates with the Wald method to provide 95% confidence intervals (*N*_*trials*_ = 1000):

$$\begin{array}{c} \hat{p}\pm 1.96\sqrt{\frac{\hat{p}\left(1-\hat{p}\right)}{N_{trials}}} \end{array}$$ (9)

In order to systematically characterize how different parameters of our training protocol affect the sensitivity of the resulting network to noise, we estimated *σ*_50_ as the value of noise variance *σ* for which the probability of correctly recalling a given sequence is 0.5. Finding such *σ* is an instance of the Stochastic Root Finding Problem [89]. To estimate this we used the naive bisection algorithm for deterministic functions by using the averages as estimates of the actual values. We stopped the algorithm as soon as the success rate corresponding to our estimate of *σ*_50_ was contained in the Wald confidence interval given in Eq 9. We find that our method was consistently able to find solutions to the root finding problem (see S1 Fig).

## Supporting information

S1 Fig Calibration of *σ*_50_ estimation. (A) two success rate vs noise profiles for *T*_*p*_ = 50 *ms* and *T*_*p*_ = 200 *ms*. The values of *p*_50_ are annotated for reference. (B-F) We show the values of *p*_50_ obtained after running the algorithm in Fig 8. For every value we see that the values of the found roots (*p*_50_, blue lines) was within confidence bounds (here blue shaded) of the expected value (0.5, horizontal lien in gray).
(PDF)

S1 Appendix Complete treatment of the persistence time. (PDF)

S2 Appendix Sequence transition as probabilistic inference. (PDF)
