# Supplementary figures and images for: Probabilistic associative learning suffices for learning the temporal structure of multiple sequences
Source: PLoS One. 2019 Aug 1;14(8):e0220161. doi: 10.1371/journal.pone.0220161 (PMC6675053; doi:10.1371/journal.pone.0220161)

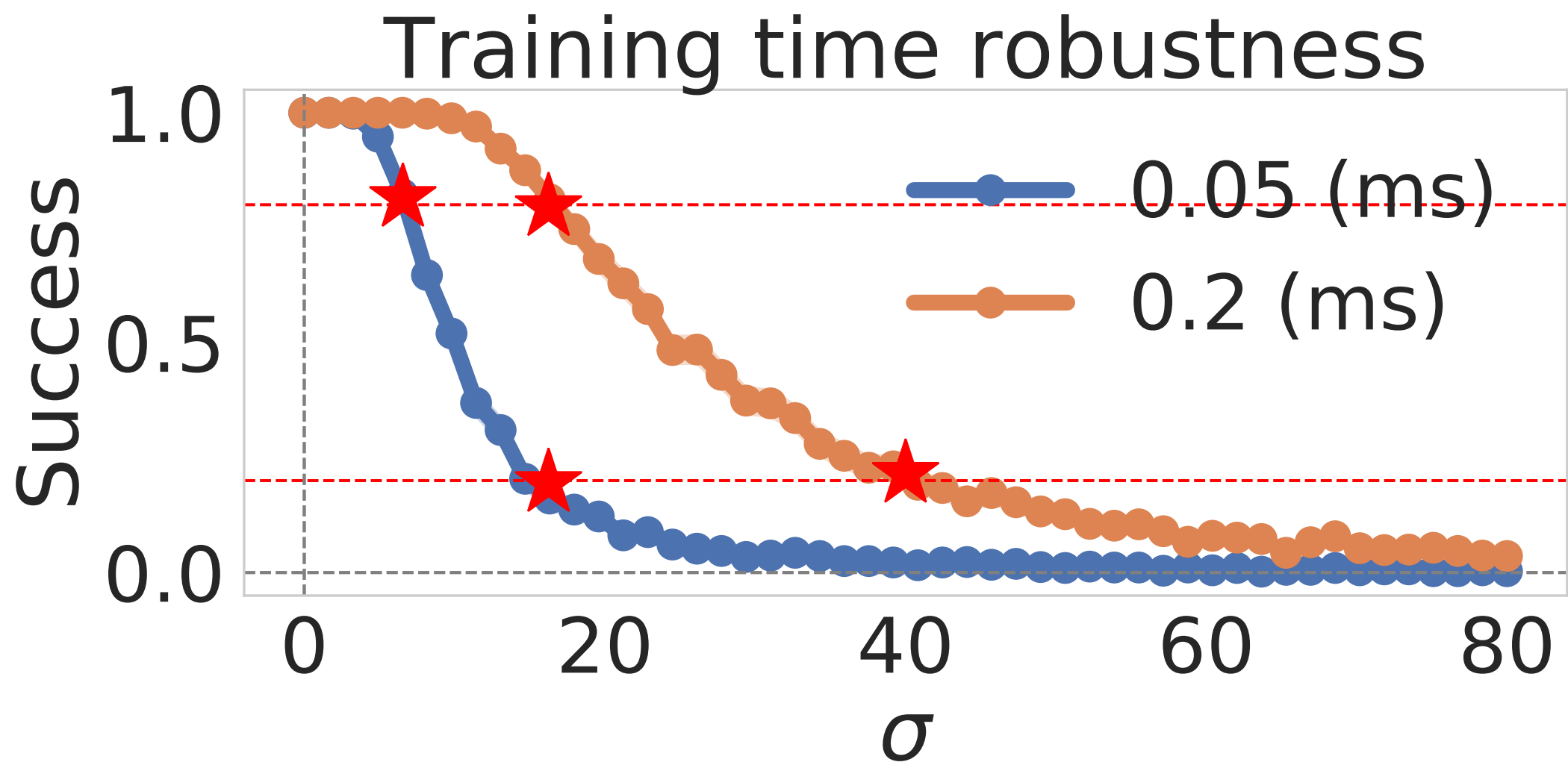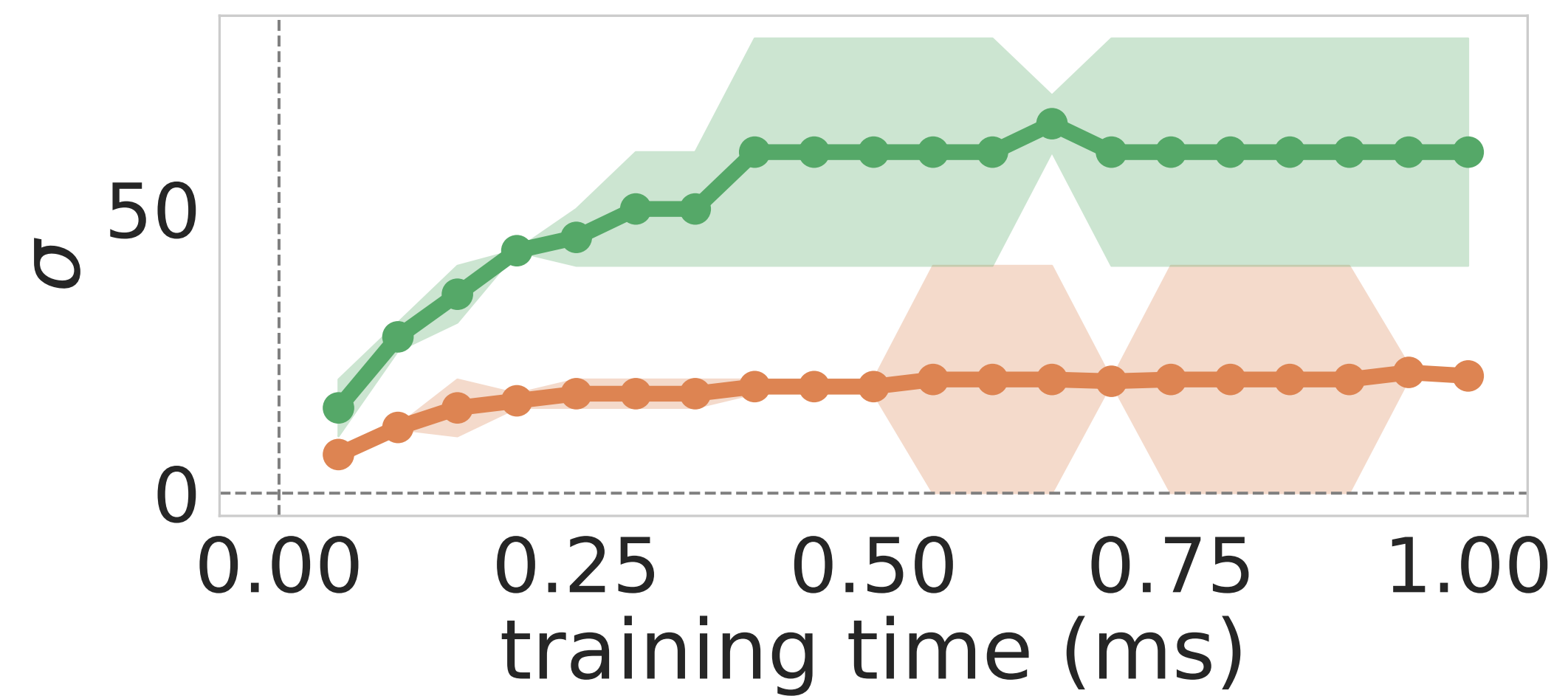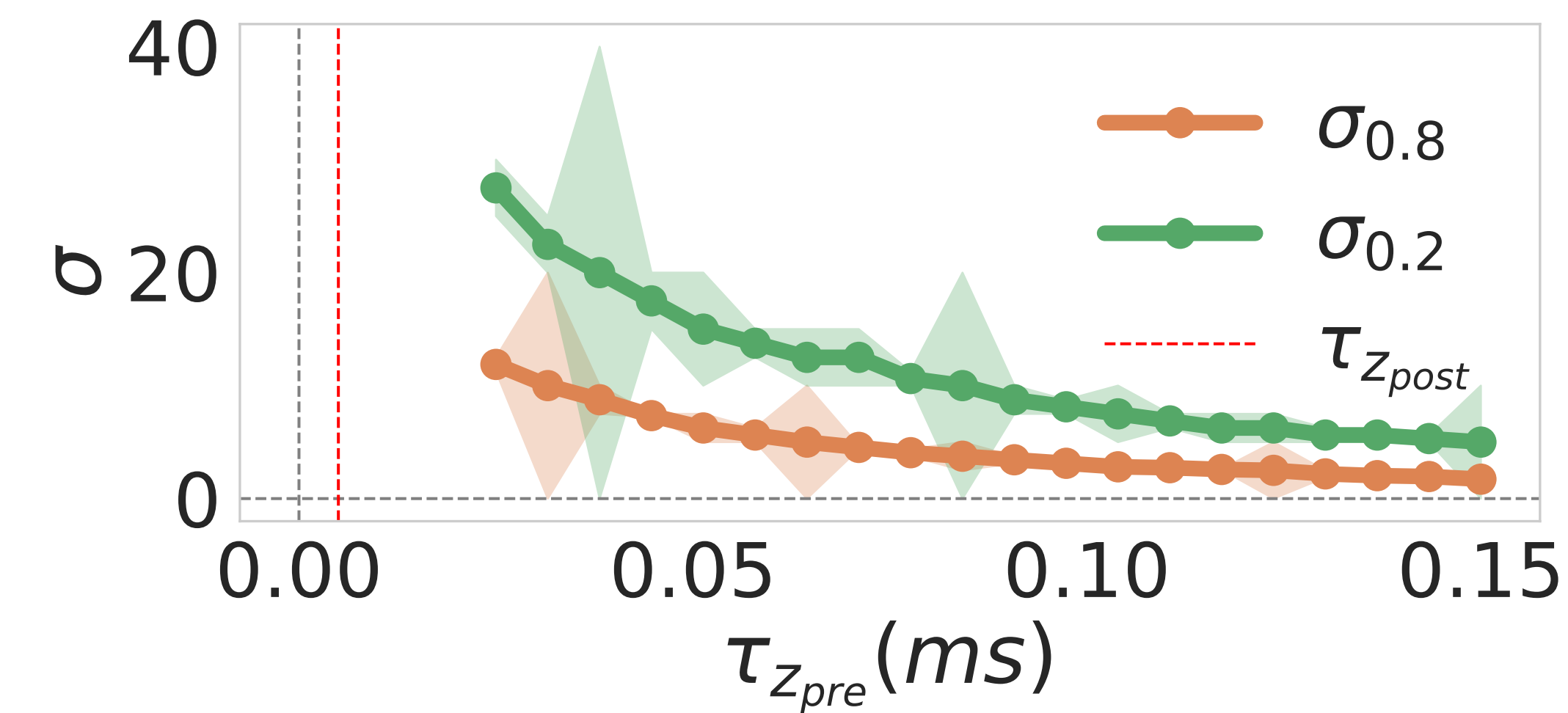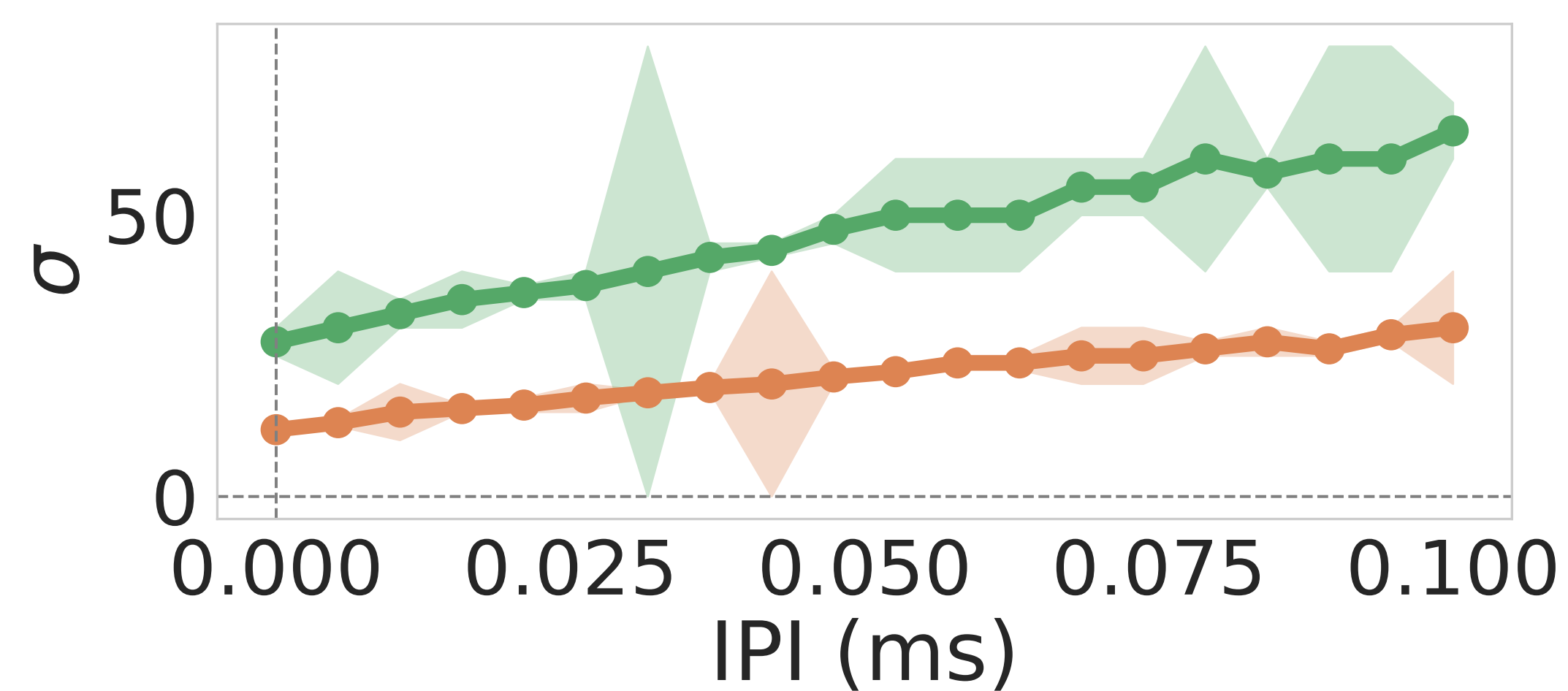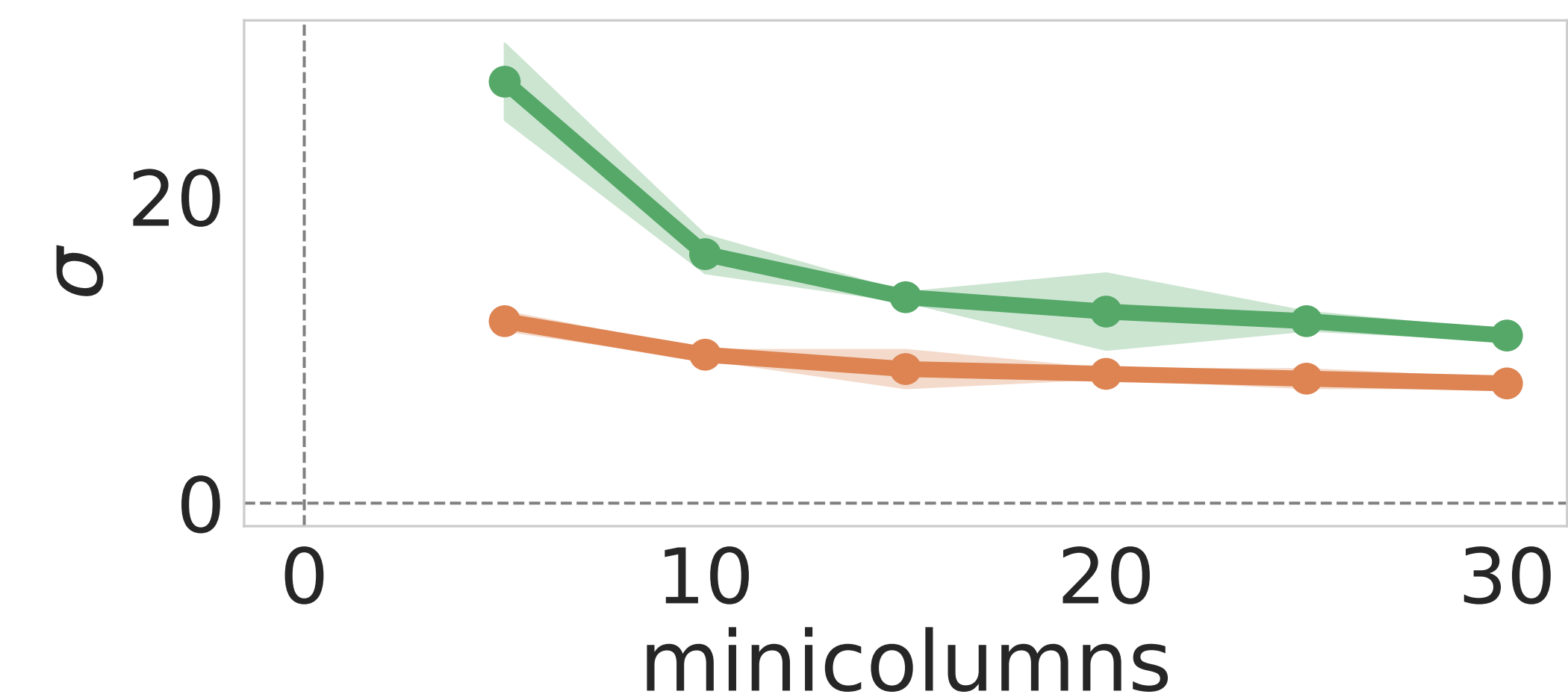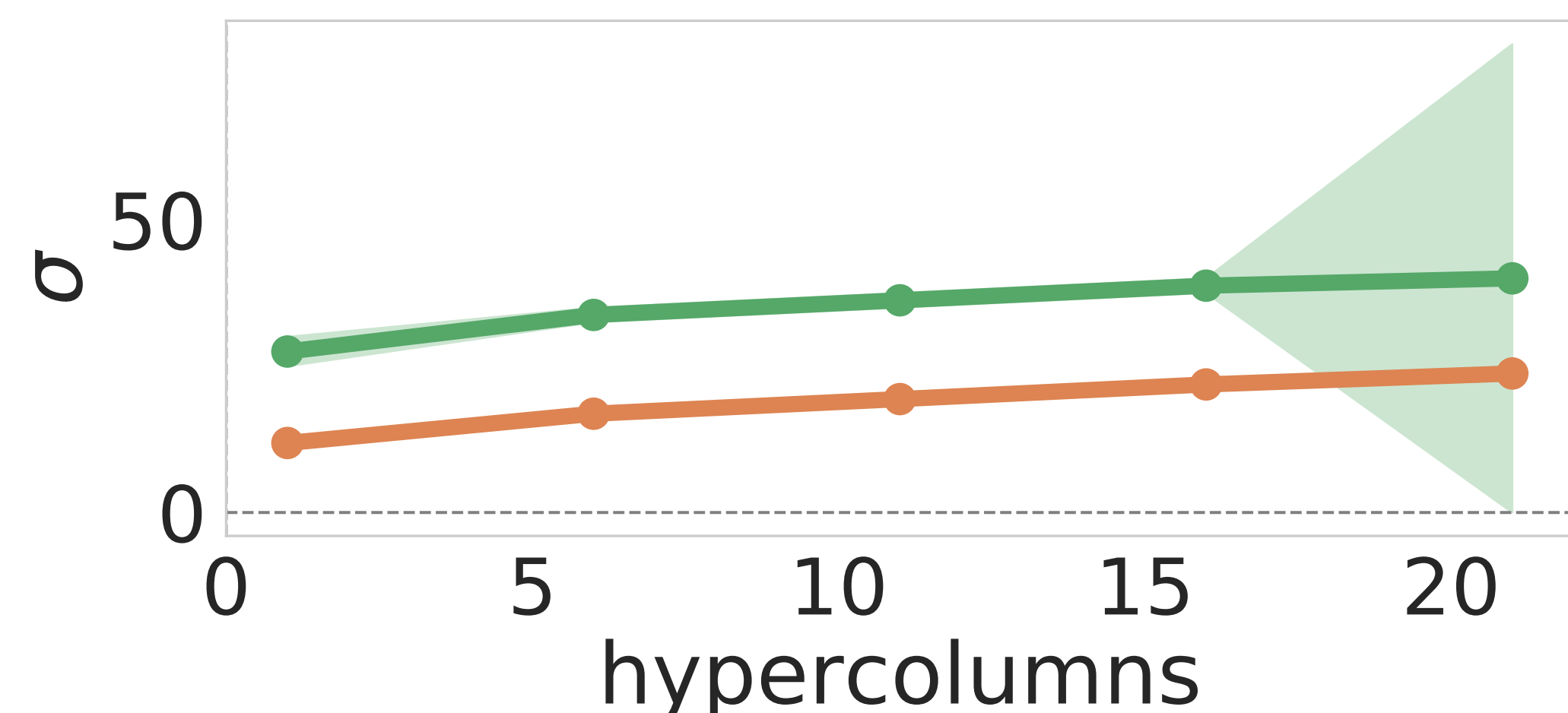

Supplement: S1 Fig — (A) two success rate vs noise profiles for Tp = 50 ms and Tp = 200 ms. The values of p50 are annotated for reference. (B-F) We show the values of p50 obtained after running the algorithm in Fig 8. For every value we see that the values of the found roots (p50, blue lines) was within confidence bounds (here blue shaded) of the expected value (0.5, horizontal lien in gray). (PDF) [file pone.0220161.s001.pdf]
