# Supplementary material for: Probabilistic associative learning suffices for learning the temporal structure of multiple sequences
Source: PLoS One. 2019 Aug 1;14(8):e0220161. doi: 10.1371/journal.pone.0220161 (PMC6675053; doi:10.1371/journal.pone.0220161)
Supplement: S1 Appendix — (PDF) [file pone.0220161.s002.pdf]

S1 Appendix. Complete treatment of the persistence time.

To characterize the transition from pattern m to pattern n (standing for P_m and P_n in the text) in the units belonging to hypercolumn j we need to calculate the difference in their respective currents $s_{mn_j}(t) = s_{n_j}(t) - s_{m_j}(t)$ as the persistence time is given by the root of the equation $s_{mn_j}(t) = 0$. Here we have adopted the convention that m_j and n_j give the index of the unit belonging to pattern m and n in the hypercolumn j respectively. To obtain a solution for $s_{mn_j}(t)$ we solve the resulting differential equation with the method of undetermined coefficients.

$$s_{mn_j}^{\infty} = \frac{1}{H}\sum_{i}^{H} \Delta w_{m_i n_j} + \Delta\beta_{mp_j} + \Delta I_{mn_j} - g_a \delta_{m_j n_j}$$

$$\begin{aligned} s_{mn_j}(t) &= s_{mn_j}^{\infty} + g_a \left(\frac{1 - a_{m_j}(0) + a_{n_j}(0)}{1 - \frac{\tau_s}{\tau_a}} \right) e^{-\frac{t}{\tau_a}} \\ &+ \left(s_{mn_j}(0) - s_{mn_j}^{\infty} + g_a \left(\frac{1 - a_{m_j}(0) + a_{n_j}(0)}{1 - \frac{\tau_s}{\tau_a}} \right) \right) e^{-\frac{t}{\tau_s}} \end{aligned} \quad (1)$$

Where $\Delta w_{m_i n_j} = w_{m_i m_j} - w_{m_i, n_j}$ are the weights of the differential input coming to hypercolumn j from hypercolumn i, $\Delta\beta_{mn_j} = \beta_{m_j} - \beta_{n_j}$ is the local (same hypercolumn) differential in intrinsic excitability and $\Delta I_{mn_j} = I_{m_j} - I_{n_j}$ is the differential external input to the units belonging to m and n in the hypercolumn j. Note that the sum is only over each of the (H) units active in each hypercolumn.

When pattern m becomes active the units that belong to it start experiencing intrinsic adaptation through the terms a_{m_j} and, in consequence s_{m_j} starts decreasing. It follows that the current s_{m_j} becomes smaller than s_{n_j} at some point in time and the transition occurs. We denote such time as $T_{mn_j}^{per}$ to emphasize that we are talking about transition from pattern m to n in hypercolumn j. Formally, this time can be found by setting $s_{mn_j}(t)$, above equal to 0. If we disregard the short-term fluctuations of the term $e^{-\frac{t}{\tau_s}}$ we obtain the following expression:

$$T_{mn_j}^{per} = \tau_a \log\left(\frac{1 - \Delta a_{mn_j}(0)}{1 - B_{mn_j}} \right) + \tau_a \log\left(\frac{1}{1 - \frac{\tau_s}{\tau_a}} \right) \quad (2)$$

Where $B_{mn_j} = \frac{\frac{1}{H}\sum_i^H \Delta w_{m_i n_j} + \Delta\beta_{mn_j} + \Delta I_{mn_j}}{g_a}$ and $\Delta a_{mn_j}(0) = a_{m_j}(0) - a_{n_j}(0)$. Note that the previous presence of adaptation in the unit of pattern m, $a_{m_j}(0)$, decreases the persistence time and previous presence of adaptation in the unit of pattern n, $a_{n_j}(0)$, has the opposite effect.

In the case of multiple hypercolumns there is a value of B_{mn_j} for every hypercolumn j determining how fast the transition happens at that hypercolumn. As a matter of fact, the transition happens only if all the B_{mn_j} are less than 1. To see this, note that if B_{mn_j} is less than 1 the value of $s_{mn_j}^{\infty} = g_a B_{mn_j} - g_a$, the steady state of the difference in support values, is negative implying that the adaptation is strong enough to induce a transition. The transition is fast for B_{mn_j} close to 0 and slow for B_{mn_j} equal to 1 (modified by memory effects of the adaptation). These two effects combined give the order in which the units of a pattern belonging to different hypercolumns undergo transition. However, the exact timings at which the transitions happen are modified after the first transition takes place; this is because the currents that the rest of the

units of pattern n receive (the ones in the other hypercolumns) are modified as well. In general this have the effect of accelerating the transition of the other units belonging pattern n. By taking this modifications into account we can derive conditions for the modification of T_{per} in the remaining hypercolumns after a transition in hypercolumn k has happened (up to time differences in the order of τ_s due to membrane capacitance effects):

$$T^{per}_{mn_l} = \tau_a \log\left(\frac{1 - \Delta a_{mn_l}(T^{per}_{mn_k})}{1 - B^{new}_{mn_l}}\right) + \tau_a \log\left(\frac{1}{1 - \frac{\tau_s}{\tau_a}}\right) \tag{3}$$

$$B^{new}_{mn_l} = B^{old}_{mn_l} - \frac{1}{g_a H}\left(\underset{repeated}{\Delta w_{m_k n_l}} + \underset{back}{\Delta w_{n_k m_l}}\right) \tag{4}$$

$$\Delta a_{nm_l}(T^{per}_{nm_k}) = 1 - (1 - \Delta a_{nm_l}(0))e^{-\frac{T^{per}_{nm_k}}{\tau_a}} \tag{5}$$

The $B^{new}_{nm_l}$ term is now reduced by the lost self-excitatory current from unit m_k, $w_{m_k m_l}$ (we also subtract the lost of the feed-forward current $w_{m_k n_l}$) This reduction is reflected in the subtraction of the term $\Delta w_{m_k n_l} = w_{m_k m_l} - w_{m_k n_l}$. The now activated unit n_k induces a backward current: $w_{n_k m_l}$. Also there is a recurrent current helping to fix the m_l unit coming from hypercolumn k, $w_{n_k n_l}$. These contributions are reflected in the addition of the terms $w_{n_k m_l} - w_{n_k n_l}$ to the expression above which we write with a minus sign as: $\Delta w_{n_k m_l} = w_{n_k n_l} - w_{n_k m_l}$. The overall effect of these new currents (mainly coming from the backwards negative current $w_{n_k m_l}$) is to reduce the value of $B^{new}_{mn_l}$ with respect to $B^{old}_{mn_l}$ thus effectively hastening the transition. Moreover, as time passes, the adaptation current tends to become larger in the units that are activated and smaller in the inactive units, which also contributes to acelerate the transition. This effect is reflected in the quantity $\Delta a_{mn_l}(t)$ becoming closer to 1. We can use this effect iteratively to calculate the values of $T^{per}_{mn_l}$ for every hypercolumn using the formula above recursively.

To derive conditions for synchronous transition we notice that if the term inside the logarithm becomes less than 1 it means that the quantity becomes negative implying instantaneous transition. This is accomplished when the following condition is satisfied:

$$B^{new}_{mn_l} < \Delta a(T^{per}_{mn_k}) \tag{6}$$

As long as there is a hypercolumn for which this value is satisfied the transition takes place there. This in turn, means that the values of B have to be updated again (making them smaller) rendering a transition in the other hypercolumns more likely. This creates a cascade effect where the latter transitions happen overwhelmingly faster than the first ones.

Please note that while this provides us with transition times for all the hypercolumns between two patterns, it does not guarantee that the aforementioned transitions take place. It is still possible that other values of $T^{per}_{mn_l}$ are smaller and those are the transitions that in fact occur
