# Supplementary material for: Probabilistic associative learning suffices for learning the temporal structure of multiple sequences
Source: PLoS One. 2019 Aug 1;14(8):e0220161. doi: 10.1371/journal.pone.0220161 (PMC6675053; doi:10.1371/journal.pone.0220161)
Supplement: S2 Appendix — (PDF) [file pone.0220161.s003.pdf]

S2 Appendix. Sequence transition as probabilistic inference.

In this appendix we show how a neural network trained with the BCPNN rule above implements an exploration of its memory patterns that follows the transition probabilities presented during training. In a way this makes the BCPNN a local learning rule for a Markov Chain structure. Again, we adopt the convention that m_j and n_j give the index of the unit belonging to pattern m and n in the hypercolumn j, respectively . Assuming that the pattern m is currently active in the network in, we then solve the differential equation for the support value $s_{n_j}(t)$ (unit belonging to pattern n in hypercolumn j) and write its steady-state value as:

$$s_{n_j}^{\infty} = \beta_{n_j} + \frac{1}{H}\sum_{i}^{H} w_{m_i n_j} - g_a \delta_{m_j n_j} \tag{1}$$

$$= s_{n_j}^{*} - g_a \delta_{m_j n_j} \tag{2}$$

Note again that the sum is only over H as there is only one unit active in each of the H hypercolumns. To frame the problem in a probabilistic fashion we remember the definitions of the weights and biases in Eq 8 in the main document and simplify:

$$s_{n_j}^{*} = \beta_{n_j} + \frac{1}{H}\sum_{i}^{H} w_{m_i n_j} \tag{3}$$

$$= \log p_{n_j} + \frac{1}{H}\sum_{i}^{H} \log\left(\frac{p_{m_i n_j}}{p_{m_i} p_{n_j}}\right) \tag{4}$$

$$= \log p_{n_j} + \frac{1}{H}\sum_{i}^{H} \log\left(\frac{p_{m_i n_j}}{p_{m_i}}\right) - \frac{H}{H}\log p_{n_j} \tag{5}$$

$$= \frac{1}{H}\sum_{i}^{H} \log\left(\frac{p_{m_i n_j}}{p_{m_i}}\right) \tag{6}$$

We have discussed previously that a transition occurs when the differences in the support values $s_{mn_j}^{*} = s_{m_j}^{*} - s_{n_j}^{*}$ becomes negative. How fast the transition happens, on the other hand depends on the width of this gap, as it is exactly the size that the adaptation current has to breach before the transition occurs. We can calculate this quantity as:

$$s_{mn_j}^{*} = \frac{1}{H}\sum_{i}^{H} \log\left(\frac{p_{m_i m_j}}{p_{m_i}}\right) - \frac{1}{H}\sum_{i}^{H} \log\left(\frac{p_{m_i n_j}}{p_{m_i}}\right) \tag{7}$$

$$= \frac{1}{H}\sum_{i}^{H} \log\left(\frac{p_{m_i m_j}}{p_{m_i}}\frac{p_{m_i}}{p_{m_i n_j}}\right) = \frac{1}{H}\sum_{i}^{H} \log\left(\frac{p_{m_i m_j}}{p_{m_i n_j}}\right) \tag{8}$$

$$= \log\left(\prod_{i}^{H}\frac{p_{m_i m_j}}{p_{m_i n_j}}\right)^{\frac{1}{H}} \tag{9}$$

We have established than in general the difference in support values in a hypercolumn is given by the logarithm of the geometric mean of the ratio between joint and transition probabilities. If we have multiple patterns stored in the network (say n and k) then the network transitions to the one with the smallest gap (s_{mn_j} vs s_{mk_j}). In the expression above the term in the nominator does not depend on n or k, which means that the smaller gap is the one with the largest denominator ($\prod_i^H p_{m_i n_j}$ vs $\prod_i^H p_{m_i k_j}$). In short, the network "compares" the product of the transition probabilities and transition with the one with higher values.
