# Supplementary material for: Probabilistic associative learning suffices for learning the temporal structure of multiple sequences
Source: PLoS One. 2019 Aug 1;14(8):e0220161. doi: 10.1371/journal.pone.0220161 (PMC6675053; doi:10.1371/journal.pone.0220161)
Supplement: Attachment — Submitted filename: 2019-06-26(Response to the reviewers).pdf [file pone.0220161.s004.pdf]

Dear Editors and Reviewers

We are grateful for the option to reconsider our contribution for the publication in PLOS ONE.

As requested, in this letter you can find a detailed answer to the Reviewers' comments regarding our manuscript. We are grateful for the effort you poured into writing interesting and detailed points in your request and hope you find our responses convincing. Included with this letter there should be a new corrected version of the manuscript that takes into account the Reviewers' comments and a second text where the differences between the old and the new manuscript are highlighted; we refer to this text as the diff text. Besides addressing the Reviewers' points we also implemented stylistic and writing improvements to the manuscript with the aim of improving the overall quality of the presentation. Following editorial guidelines, we have confined the appendices to separate files, changed the format of our table and removed a reference to data that is not available in the manuscript.

Format: we show the reviewers' comments in **bold** and our answers follow.
=======================================
Reviewer 1:
No major or minor comments
=======================================
Reviewer 2
1) "**My main concern is that the relation to previous theoretical work in this field is insufficiently described. A number of general features of the model have been used previously by Pereira and Brunel, Veliz-Cuba et al, and others: a global inhibition/mutual inhibition mediated winner-take-all mechanism that assures the activity of a singel unit, Hebbian-like plasticity, adaptation/facilitation as a driver of sequential activation, need for overlapping activity/memory traces for learning, similar expressions relating model parameters to sequence activity length, observation that noise shortens the activation interval, and so on. However, the structure of the network here is different which does lead to important differences, such as a different - as far as I could understand - response to noise, as well as the ability to learn overlapping sequences. These similarities and differences need to be described in detail, and the advantages of this approach explained"**

To address these concerns we have included a new section in the discussion and an accompanying figure (see lines from 418-442 in the diff file). We emphasize that while other theoretical contributions use similar features (not all) and share similar concerns (persistence time, overlapping traces, pattern-overlap) our work addresses all of them systematically under the same framework with emphasis on learning on multiple sequences. We elaborate on why we believe the BCPNN learning rule provides a better foundation for the study of multiple sequence learning in the text (see lines from 418-442 in the diff file).

2) **"A number of terms are introduced, but not explained: For instance, success rate and \sigma_{50} are not defined in the text. While readers could infer their meaning, they should not be required to do so"**

Thank you for pointing this out. We have now provided a definition and a pointer to the proper section in the methods respectively when these elements are employed for the first time (see lines 278 and 286 in the diff).

3) "**The learning rule is insufficiently motivated. In the introduction that the network performs inference - inference of what? The rule itself is framed in terms of probabilities, but probabilties of what? Are these the freuqencies of events in the sequence? Is z_i z_j a measure of co-occurence? Is some independence assumed? I see that the rule is effectively Hebbian, so would it not be possible to use a straightforward Hebbian rule? If not, what would be lost? Overall, the rule was insufficiently explained and motivated, and left me confused. I just did not see the need for a Bayesian formulation, since the learning process was not described as in inference process — unless the order and lenghts of activations were being inferred by the network. If this is the viewpoint, the authors should clearly articulate it.**"

Thank you for this interesting comment, particularly in relation to a simple Hebbian learning rule. To answer this concern we have included a new Appendix S2 where we show how the transition from one pattern to another follows the transition statistics of the training set (see the new S2 appendix). The answer to the question of how the Hebbian learning rule differs from the BCPNN in terms of sequence learning is also given in the new section (3.1) of the discussion and its accompanying figure (see lines from 418-442 in the diff file)

4) "**The aim of previos rules was not only to learn sequence order, but also the timing of different events in the sequence. It was unclear to me to what extent the present model can do so. If this is possible, it should be mentioned. Also the authors should discuss in more detail the need, or lack thereof, for parameter tuning.**"

We are also thankful for this question requesting a direct comparison. To answer this we added an enhanced discussion of the learning of temporal order in our Discussion section. We argue that our model provides the same functionality as the past contributions to the literature (see lines 504-515 in the diff text). Regarding parameter tuning, we added a section discussing the issue at the beginning of our discussion (see lines 407-417 in the diff-text).

Minor comments from reviewer 2:

- I was somewhat confused about what the authors mean by “pattern”. This may be just me, but I suggest that the authors define an (activation) pattern clearly in the text and in the caption of Fig. 1 more explicitly.

We added a clarification to the definition of the pattern (see line 108 in the diff text).

- I would avoid the term “brain phenomena”
We remove the term, see changes in the abstract.

- In eqs 1 - 3, what is the base rate, beta? What is H?
Beta represents a level of intrinsic current given and is related to the overall probability of activation through log (p) (see Eq. 8). H is the number of hypercolumns, we have added this to the table of symbols with the hopeful of making the presentation clearer.

- The max rule in Eq. 2 seems biologically implausable. However, I think there shoudl be alternative ways.
We agree with the reviewer in this point. We describe possible (and more elaborate) implementations of this is a network in Section 3.2. However, we felt that this was not central to our work.

- The sentence before Eq 4 is unclear
We modified the sentence before equation 4 (see lines 149-152 in the diff text).

- Eqs 4-6 are similar to previous work, as discussed above. This should be mentioned.
We included a discussion of the use of equations 4-6 in the literature and our work (see lines 504-515 in the diff text).

- Fig 10A is a bit unclear. What happesn in the white space in the corner? What do the dashed lines mean?
We added clarification of what is going on in the corner of the parameter space in Fig10A in its caption.

I did not understand Fig. 6b and the independent learning of patterns. It appears that patterns are just randomly activated. Is this the point? If so perhaps relate to Kumar & Doiron, Nat Neuro

* We modified the caption of Fig 6 in line with the Reviewer’s comment.

- line 265 - What is success rate? What is sigma_50 below.
See response to major point 2.

- It is interesting that disambiguation is possible with complete pattern overlap. The authors describe this briefly, but a better explantaion would be appreciated.

* We elaborate more on the discussion of the disambiguation with complete overlap (see lines 394-396 in the diff text)

Overall we are grateful for the detail and thoroughness of the provided comments.

=====================
Reviewer 3

Major comments:
1) “**The network is composed of a number hypercolums which corresponds to a pool of what author call units. The authors don’t specify what actually are, are they neurons? Are they populations of neurons?”**

To address the concerns of the reviewer we have added clarification (see lines 101 in the diff text).

“What authors call dynamics of each hypercolumns is given by a max operation over the activity of the units described by Eq.2, that is at each point in time the units that received the larger current will be active (the activation variable is equal to 1) and the rest of units in the hypercolumn will be inactive (the activation variable is equal to 0). The dynamics for the current to each unit is given by Eq.1. The authors argue this max operation is equivalent to a winner-take-all dynamics (WTA) among multiple competing units in the hypercolumn. However, it is not clear to me how the recurrent connection of each hypercolumn will lead to such a WTA dynamics. In biological networks, WTA dynamics need both strong excitation and strong shared inhibition (see Wang, 2002, Neuron; Wong&Wang, 2006 JNeuroscience). The authors do not address how the network can have both WTA and sequential activity steming from the same recurrent connectivity, neither how WTA can be learned to sustain the sequential activity in their model. Therefore, the max operation seems an artificial resort or obtaining the dynamics intended. I want to be clear that my concern is not that there is WTA dynamics in the network, is that WTA dynamics is not sustained by the network connectivity, therefore the model is not self-consistent, lacking of biological plausibility. “

We appreciate the Reviewer’s reflections on this point and apologise for the lack of clarity. To alleviate the notion of WTA in our model, we should actually refer to “WTA mechanism” rather than “WTA dynamics”. In the presented model, local WTA mechanism is implemented as a max rule/operation (Eq.2), not by means of the dynamic interactions between units within each hypercolumns. We definitely agree with the Reviewer that this is not a biologically plausible implementation of the competition and have modified Section 3.2 accordingly to reinforce the point (we have implemented this in a spiking version of the BCPNN model, e.g. Fiebig and

Lansner, J. Neurosci. 2017, 37 (1) 83-96; doi.org/10.1523/JNEUROSCI.1989-16.2016). This is in fact intended for further work in the context of this abstract rate-based model. In the presented manuscript, we consider this aspect beyond the scope but acknowledge this limitation in the Discussion of the revised manuscript.

2) "I found this paper very difficult to read. I found systematically: i) lack of explanation of the basics of the model and the lack of definitions; ii) imprecise or unsubstantiated claims; iii) Claims often supported by citations to single experimental papers that might mislead to the reader. I will briefly explain these points above and provide some examples, however, I found this widespread within the manuscript."

i) "In many sections, the paper lacks both basic mathematical definitions and the explanation of the biological meaning of the component of their model. For example, it is not mentioned what is what the authors define as 'unit'. Is it a neuron? Is it a population of excitatory of inhibitory neurons? This is a crucial point to mention this since for example, different mechanisms are needed to lead a single neuron to undergo WTA dynamics than a population of neurons. In figure 6, authors show what they call 'free attractors' without defining what they are and what is the relation with what is classically understood as attractors in the field (see Hopfield, 1982 for example). In Eq. 1, What is H? I cannot find its definition in the text. In the same equation, the sum over i is over the hypercollumns or over the units, is not explained. In line 138 is mentioned the 'connectivity between patterns' what is the actual meaning of this phrase? A far I understand, the recurrent connectivity is among units. "

We appreciate that reviewer points out these issues. To address them we have made the following modifications:

- We have clarified what we mean by unit and what it represents (see line 101 in the diff text).
- We have included a definition of pattern (see line 108 in the diff text)
- We have added a proper definition of H (the number of hypercolumns). Moreover, we have added the symbol to Table 1 with the hope that it will ease and clarify the presentation.
- We have modified the expression `connectivity between the patterns' to its more precise and intended meaning of `the connectivity between the units belonging to a pattern' (see line 142 in the diff text)

ii) In line 623 is stated: 'as matter of fact the transition happens only if all the Bmni are less than 1'. However, there is not provided any proof for that statement. Line 209: 'Traces p absorve information..' what 'absorbing information' means?

To address this particular instance of the Reviewer’s concern about lack of precision, we re-wrote the expression of the steady state values of s and Bmni to a formulation which hopefully would clarify how the claim that “transitions happen only if all the Bmni are less than 1’’ follows from the definitions presented in the text (see modifications in S1 appendix, fifth paragraph).

We also removed the expression "absorption" in line 217.

“iii) In line 400 as well few other parts of the text it is argued that the WTA is a general feature in cortex supporting this claim with Douglas and Martin, Annu. Rev. Neurosci. 2004. While WTA dynamics well describe the observed neuronal dynamics in some brain regions as LIP, it is known that there are many different dynamical regimes in cortex as mixed selectivity in PFC (Rigotti et al, Nature 2013), non-normal dynamics in the primary visual cortex (current opinion neuro, Ken Miller), attractor dynamics in ITC (Pereira&Brunel, Neuron, 2018) among some examples. Therefore, this statement, repeated a couple of times throughout the text, is misleading. “

We admit being selective when citing experimental work. However, our only intention is to choose papers where the arguments and support for the modelling claims is most straightforward. At the same time, to avoid any risk for misleading the reader we are critical about these biological links to the extent where we claim the biological interpretation is justified.

To respond to this particular example mentioned by the Reviewer and to avoid misunderstanding, we have changed the notion of “WTA dynamics” into “WTA mechanism” (see diff text, overall). In fact, we refer to a computational mechanism of competition (in the spirit of lateral inhibition) and consider the WTA mechanism as a simplistic implementation. We comment on this limitation in the Discussion (section 3.2), as pointed out in our response to the first concern raised by the Reviewer.

Still, when we refer to competition in neural circuits we make a claim that it is a popular computational motif, particularly in the spirit of lateral inhibition, and in support we add extra references (see the modifications in section 3.2 in the discussion). At the same time, we absolutely agree that there are other types of dynamic regimes and their computational interpretations in cortex.

“I want to stress that the examples above are not cherry-picked, but it seems to me that i-iii types of statements are widespread across the text.”

We understand and appreciate the Reviewer’s concerns about lack of clarity, precision and specificity in some of our formulations. We have now revised the manuscript demonstrating special care for the clarity of the presentation in line with the problems enumerated by the Reviewer. We expect that in the revised manuscript these undesirable statements of type i-iii, as indicated by the Reviewer, have been sufficiently addressed.

====================================

Following editorial guidelines we removed the mentions of “data not shown”. The first was involved in the discussion of some preliminary results where the use was not appropriate (line 619) and the second was the mention of a study of the spread of the failure of the network (line 671).
